# Comprehensive annotation and characterization of planarian tRNA and tRNA-derived fragments (tRFs)

**DOI:** 10.1261/rna.077701.120

**Published:** 2021-04

**Authors:** Vairavan Lakshmanan, T.N. Sujith, Dhiru Bansal, Padubidri V. Shivaprasad, Dasaradhi Palakodeti, Srikar Krishna

**Affiliations:** 1 Institute for Stem Cell Science and Regenerative Medicine (inStem), 560065 Bangalore, India; 2 SASTRA University, 613401 Thanjavur, India; 3 National Centre for Biological Sciences (NCBS), 560065 Bangalore, India

**Keywords:** planaria, regeneration, small RNA, tRFs, tRNA

## Abstract

tRNA-derived fragments (tRFs) have recently gained a lot of scientific interest due to their diverse regulatory roles in several cellular processes. However, their function in dynamic biological processes such as development and regeneration remains unexplored. Here, we show that tRFs are dynamically expressed during planarian regeneration, suggesting a possible role for these small RNAs in the regulation of regeneration. In order to characterize planarian tRFs, we first annotated 457 tRNAs in *S. mediterranea* combining two tRNA prediction algorithms. Annotation of tRNAs facilitated the identification of three main species of tRFs in planarians—the shorter tRF-5s and itRFs, and the abundantly expressed 5′-tsRNAs. Spatial profiling of tRFs in sequential transverse sections of planarians revealed diverse expression patterns of these small RNAs, including those that are enriched in the head and pharyngeal regions. Expression analysis of these tRF species revealed dynamic expression of these small RNAs over the course of regeneration suggesting an important role in planarian anterior and posterior regeneration. Finally, we show that 5′-tsRNA in planaria interact with all three SMEDWI proteins and an involvement of AGO1 in the processing of itRFs. In summary, our findings implicate a novel role for tRFs in planarian regeneration, highlighting their importance in regulating complex systemic processes. Our study adds to the catalog of posttranscriptional regulatory systems in planaria, providing valuable insights on the biogenesis and the function of tRFs in neoblasts and planarian regeneration.

## INTRODUCTION

Transfer RNAs (tRNAs) canonically recognize the triplet codons on the mRNA, thereby delivering appropriate amino acids to the growing polypeptide chain during protein synthesis. Emerging studies have identified tRNAs as a source for a new heterogeneous class of small RNAs called tRNA-derived fragments (tRFs). Though fragments from tRNAs were observed as early as 1970s, their physiological relevance remained largely unexplored until recently (Speer et al. 1979). In recent years, several species of tRFs have been identified and shown to be conserved across the three domains of life. Based on the region of tRNAs from which these small RNAs are processed, tRFs can be categorized as tRNA derived small RNAs (tsRNAs) or tiRNA/tRNA halves (usually 30–35 nt) long and other shorter (<30 nt) fragments. There are many species of shorter tRFs such as tRF-5s (from the 5′ arm of the tRNA), itRFs (intermediate tRFs), tRFs-3s (that correspond to the 3′ arm of the tRNA). Most of our understanding of tRFs is in the limited context of cell culture systems. Functionally, the different classes of tRFs regulate a multitude of cellular processes through diverse regulatory mechanisms (Keam and Hutvagner 2015). tRNA-halves were first identified in Tetrahymena (Lee and Collins 2005). Studies from several groups have since shown an active role for tRNA-halves (or tiRNAs) under several cellular stress conditions, ascribing a role for these small RNAs in regulating translation (Thompson et al. 2008; Ivanov et al. 2011; Lyons et al. 2016). tsRNAs have also been shown to be abundantly expressed in sperm acting as paternal epigenetic factors, thereby contributing to intergenerational inheritance (Chen et al. 2016; Sharma et al. 2016b; Sarker et al. 2019). Using heterologous models of stem versus differentiating states, we previously showed that tsRNAs play an important role in regulating translation and transcript stability of specific transcripts during cell state transitions (Krishna et al. 2019b). Further, functional characterization of tRF-5s in human embryonic stem cells revealed an important role for these small RNAs in stem cell differentiation (Guzzi et al. 2018). tRF-3s have been shown to regulate gene expression via its association with Argonaute (Ago) complex, acting similar to miRNAs (Kumar et al. 2014; Luo et al. 2018), by releasing complex secondary structure within mRNAs, thereby facilitating translation (Kim et al. 2017), or by binding to promoter binding sites of retrotransposons and preventing their transcription (Schorn et al. 2017). Other tRNA-derived fragments have been shown to suppress oncogenic transcripts by scavenging RNA binding proteins such as YBX1 (Goodarzi et al. 2015). Given these findings, tRFs are likely to have significant impact on biological processes such as development or regeneration that operate at the level of a whole organism. This study aims to place tsRNAs in the context of these processes and examines the potential roles of tsRNAs during these events.

Freshwater planarians are flatworms primarily known for their remarkable ability to regenerate any lost tissue. This ability of the planarians to regenerate is mainly attributed to the specialized of adult stem cells called neoblasts (Reddien et al. 2005; Palakodeti et al. 2008; Wagner et al. 2011). Regeneration in planarians occurs in a sequence of cellular events such as wound closure and healing, proliferation and differentiation of neoblasts, and patterning of cells to develop proportionate organs. These events are controlled by underlying gene regulatory networks that spatially and temporally control the expression of specific genes thus orchestrating a coordinated regeneration process (Wenemoser et al. 2012; Reddien 2018). Planarians possess several gene regulatory programs that are critical for neoblast function and regeneration (Wagner et al. 2011; Solana et al. 2012; Wenemoser et al. 2012; Sasidharan et al. 2013a; Lakshmanan et al. 2016; Bansal et al. 2017; Krishna et al. 2019a). Small RNAs are one of the key regulators of gene expression. Previous studies have comprehensively characterized the expression of several microRNAs (miRNAs) during planarian regeneration (Sasidharan et al. 2013a). Knockdown of the *miR-124* family of miRNAs, resulted in the mispatterning of the brain and central nervous system during regeneration (Vidyanand et al. 2017). However, our understanding of small RNAs in planarians and regeneration are largely limited to miRNAs and piRNAs. Identification of tRFs in planarians has not been possible due to the lack of a comprehensive annotation of tRNAs.

In the present study, we annotate tRNAs in planarians combining two different tRNA prediction algorithms. Our prediction of tRNAs facilitated the identification of tRNA-derived fragment pools in planarians revealing three main tRF species in planarians—tRF-5s, itRFs, and 5′-tsRNAs. Bioinformatic analysis of these small RNAs from sequential transverse sections uncovered diverse spatial expression patterns for these small RNAs across the planarian body indicating body-wide functional relevance. Further, analysis of the previously published small RNA data set during planarian anterior and posterior regeneration, identified tRFs enriched during various stages of regeneration, highlighting a crucial role for these small RNAs in regulating the various events during regeneration. Lastly, using the existing SMEDWI pulldown data, we observed that 5′-tsRNAs interact with all the SMEDWI proteins in planaria, thus offering a possible biogenesis and functionality for these small RNAs. Further, sequence analysis of itRFs revealed an enrichment for “U” at the first base hinting at processing events similar to miRNAs. Knockdown of genes involved in the miRNA pathway (*Dicer, Ago1*, and *Ago2*) revealed the possible involvement of AGO1 in the maintenance of the itRF population. Our study, for the first time, identifies a novel class of small RNAs in planarians thus expanding the posttranscriptional regulatory systems that govern stem cell function and regeneration.

## RESULTS

### Annotation of tRNAs and codon usage in planarians

**FIGURE 1.**
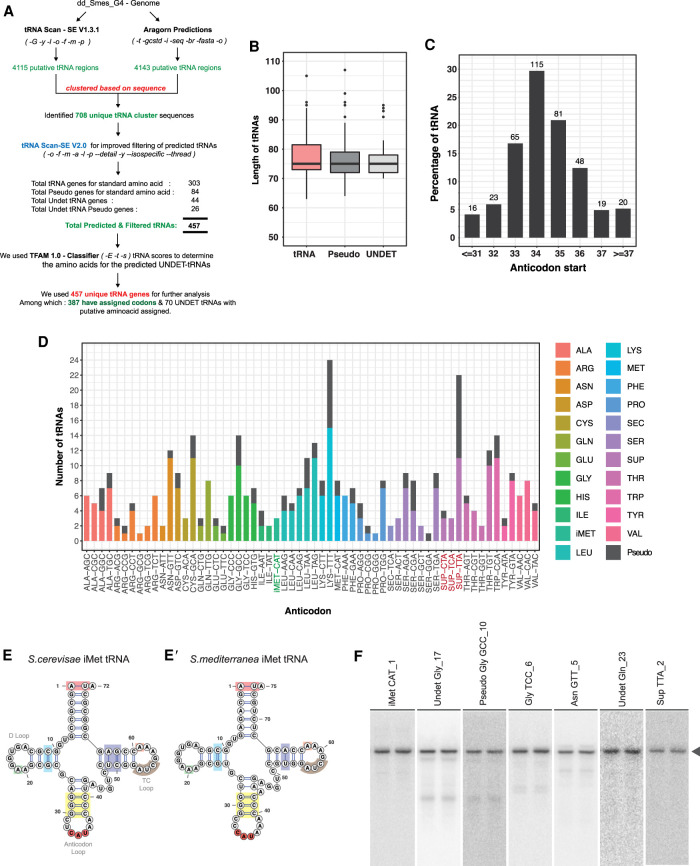
tRNA annotations in planaria. (*A*) Bioinformatic pipeline used to annotate planarian tRNAs. Two different prediction algorithms were used to identify a confident set of 457 tRNAs in *S.mediterranea*. (*B*) Median lengths of tRNA identified in *S. mediterranea*. (*C*) Bar graph depicting the position of anticodons across planarian tRNAs. (*D*) The gene copy numbers of all the identified planarian tRNAs across *S. mediterranea* genome. (*E*) Conserved features of i-Met tRNAs identified in planaria as compared to the *S. cerevisae* iMet tRNA. The colors indicate the conserved sequence signatures. (*F*) Northern hybridization blot validation of predicted tRNAs (shown in duplicates). The arrow points to the tRNA band.

To predict tRNA genes in *Schmidtea mediterranea*, we used tRNA prediction algorithms tRNAScan-SE and Aragorn to scan the dd_Smes_G4 (Grohme et al. 2018) version of the genome. tRNAScan-SE predicted 4115 putative loci while Aragorn predicted 4143 loci across planarian genomes (Fig. 1A). Sequences predicted by both programs were clustered and overlapped using CD-HIT (>90% sequence similarity) (Li and Godzik 2006b). Clustering these predictions narrowed down the tRNAs to 708 unique sequences (Fig. 1A). Further filtering these 708 sequences using tRNAScan-SE (v2.0), an improvised predictive algorithm and classifier, resulted in the prediction of 457 unique tRNA genes in planrians (Chan and Lowe 2019). The identified 457 tRNA genes were classified into three categories (Fig. 1A): (i) standard tRNA genes, which code for the standard 20 amino acids, (ii) UNDET tRNAs that are potential tRNA genes for which the programs were unable to assign a triplet codon confidently, (iii) pseudo tRNA genes that are derived from tRNAs with point mutations, insertions or deletions. The UNDET tRNAs predicted in our analysis were resolved further using TFAM (Tåquist et al. 2007) to assign the amino acids these tRNAs could code for based on sequence conservation with known tRNAs across all organisms. These 457 tRNA genes were represented across 1372 loci on the planarian genome, with Lysine having the highest number of tRNA genomic loci (104 loci for 32 tRNA genes, Supplemental Fig. S1A). The predicted planarian tRNAs had a median length of 75 nt with less variation across the three categories of tRNAs (Fig. 1B) comparable to those observed across eukaryotes (with a median length around 72/73bp) (Supplemental Fig. S1B). Analysis of the anticodon positions revealed that ∼30% of planarian tRNA anticodons originate at the 34th nt on the tRNA (Fig. 1C). Sequence analysis of the 457 predicted planarian tRNA sequences revealed high sequence conservation between the tRNAs carrying the same amino acids (Extended Supplemental 1)

Among the 457 tRNAs, 347 sequences code for the standard 20 amino acids (including UNDET tRNAs) and 110 sequences were classified as pseudogenes (Fig. 1D; Supplemental Fig. S1A,C; Supplemental Table S1). The total number of tRNA genes predicted is comparable with well annotated species (human, mouse, chick, rat, and fly) (Supplemental Fig. S2A). From our stringent prediction, we identified tRNA genes carrying anticodons against 54 standard codons out of the 61 total codons (Fig. 1D; Supplemental Fig. S1C). Among the tRNAs coding for methionine, we were able to identify three initiator methionine tRNAs (tRNA iMet-CAT) in the *S. mediterranea* genome, based on certain conserved sequence features that exist across all the eukaryotic iMet-tRNAs (Fig. 1E,E′). Further, we also identified selenocysteine and three suppressor tRNAs (SUP-CTA, SUP-TCA, and SUP_TTA) in *S. mediterranea*. Suppressor tRNAs are a class of tRNAs that can recognize stop codon (Eggertsson and Soll 1988; Hatfield et al. 1990). This class of tRNAs alleviates premature termination of protein synthesis due to mutations in the standard tRNA anticodon that results in a stop-codon (Eggertsson and Soll 1988; Hatfield et al. 1990). To validate our prediction of tRNAs, we performed northern hybridizations for candidate tRNAs belonging to the different tRNA categories (iMet-CAT, UNDET-Gly_17, UNDET-Gln_23, Pseudo Gly-GCC, Asn-GTT, and SUP-TTA_3). Northern blots revealed the expression of all the five tested tRNAs evidenced by a prominent tRNA band (Fig. 1F). Collectively, our prediction pipeline identified 457 tRNAs genes in the planarian genome, which exhibited sequence signatures and characteristics conserved across organisms.

Studies across metazoans have shown that the 64 codons are used at different capacities and these preferences vary across different biological contexts (Hiraoka et al. 2009; Bazzini et al. 2016; Harigaya and Parker 2017; Wu and Bazzini 2018; Wu et al. 2019). One of the evolutionary determinants of codon use, and thereby translational efficiency, in organism is the abundance of tRNAs (Higgs and Ran 2008). Since determining the tRNA abundance has been difficult owing to the high degree of modifications on tRNA, efforts to understand tRNA-codon relations have used tRNA gene copy numbers as a proxy (Percudani et al. 1997; Duret 2000; Du et al. 2017). In order to study the relation between the identified tRNA genes and the codon usage in *S. mediterranea*, we first calculated the codon usage using recently annotated transcriptomes (Rozanski et al. 2019). Codon frequency analysis revealed Lys-AAA, Asn-AAT, and Glu-GAA to be the most used triplet codon across the planarian transcriptome (>50% codon frequency per 1000 codons) (Supplemental Fig. S2B; Supplemental Table S2). In agreement, Lys-TTT tRNA (the cognate pair for AAA codon) exhibited the highest tRNA gene copy number, while other high gene copy number tRNAs (such as Asn-GTT, Gly-GCC, and Val-CAC) showed inverse correlations with its cognate codon usage frequency (Supplemental Fig. S2B). However, correlation between the planarian isoaccepting-tRNA gene copy number, and amino acid usage revealed a positive linear relationship with a correlation of 0.54, comparable to those observed across all three domains of life (Supplemental Fig. S2C; Du et al. 2017). Probing these dynamics might shed more light in understanding the mechanism that regulates translation in planarians.

#### tRNA-derived fragments (tRFs) in planarians

**FIGURE 2.**
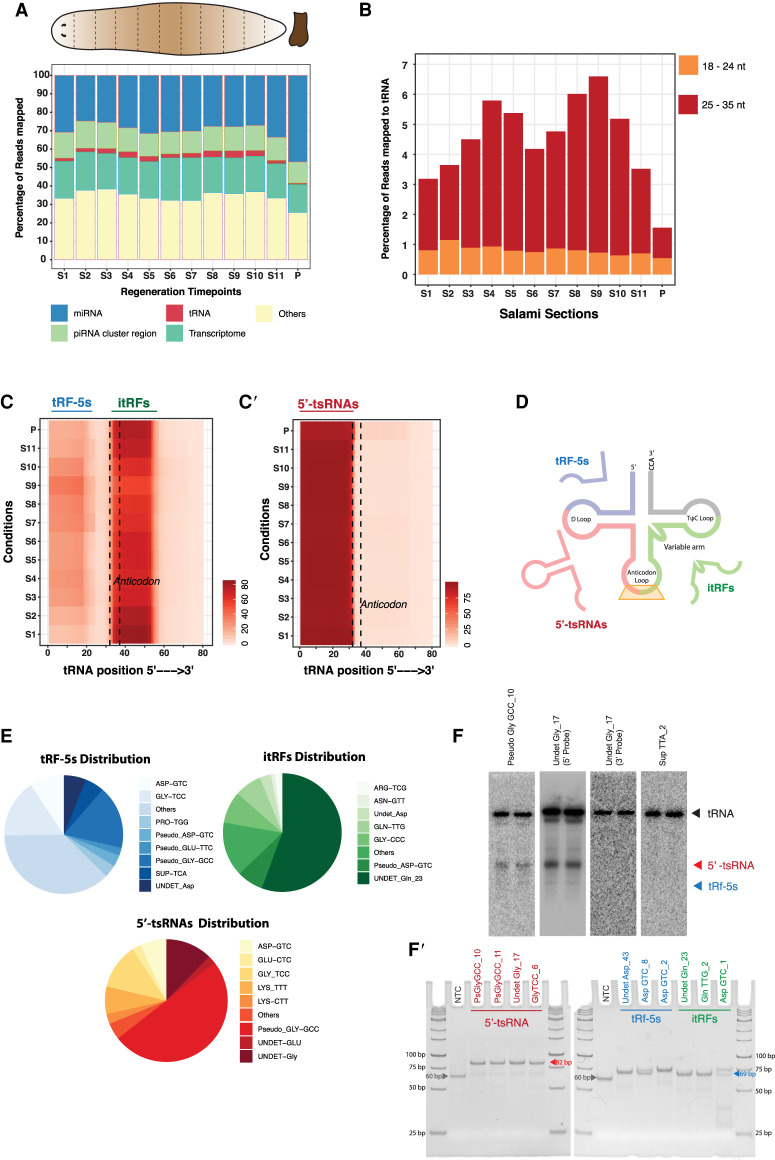
Identification of tRNA-derived fragments across planarian body axis. (*A*) Percentage distribution of all the identified small RNAs across the sections of planarian body. (*B*) Percentage distribution of small RNA reads mapping to tRNAs across planarian body sections. Small RNA reads are segregated into 18–24 nt and 25–35 nt. (*C*) Per base coverage of 18–24 nt reads across parent tRNA length. (*C*′) Per base coverage of 25–35 nt reads across parent tRNA length. (*D*) Pictorial depiction of the three tRNA-derived fragments identified in this study. (*E*) Pie chart depicting the percentage distribution of 5′-tsRNAs, tRF-5s, and itRFs in planaria. (*F*) Northern blots of top enriched 5′-tsRNAs (pseudo-GlyGCC and UNDET Gly-17). As controls, Sup-TTA (which does not generate 5′-tsRNAs) was probed in addition to probing the 3′ regions of UNDET Gly-17 to detect the presence 3′ tRFs. The two lanes represent replicates. (*F*′) Stem–loop RT-PCR validation of top planarian 5′-tsRNA, tRF-5s, and itRFs run on 10% PAGE. The gray arrowhead points to the product without any insert (∼60 bp). The red arrowhead and blue arrowhead point to sizes of tsRNA (∼80 bp) and tRF (∼70 bp) amplicons, respectively.

Using our planarian tRNA annotations, we sought to identify the tRNA-derived fragments. To obtain a holistic understanding of these small RNAs in planarians, we analyzed our previously published planarian small RNA data (Sasidharan et al. 2013a). Initially, reads from planarians (intact whole animal) were mapped to different databases, including our annotated tRNA sequences. Although miRNAs and piRNAs were the most dominant small RNA species (20.6 and 17.2%, respectively), 2.04% of reads (0.21 million reads of the 10.3 million total reads mapping to genome) mapped to our newly annotated tRNA database (Supplemental Table S3). Encouraged by these observations and in order to mine tRFs with greater sequencing depth in planarians, we sectioned planarians into 12 pieces (11 sequential sections from head to tail with pharynx as the 12th part), a procedure termed as salami sectioning (Stückemann et al. 2017). Deep sequencing of small RNAs from each of these individual sections was performed. Comparison of the tRFs from the whole animal to the averaged reads of the salami section (a proxy for the whole animal) revealed a high correlation (*R*^2^ = 0.93), highlighting the robustness of our data set (Supplemental Fig. S3A). Subsequently, we mapped 18–35 nt reads obtained from salami sections to miRNAs, piRNAs, tRNAs, etc. (Fig. 2A). The overall small RNA (18–35 nt) reads that mapped to our annotated tRNAs ranged from 0.8% to 3.91% across the salami sections (Supplemental Table S3). Though the reads mapping to tRNAs represent only a small fraction, their individual expression levels were comparable to some of the highly expressed planarian miRNAs (Supplemental Fig. S3B).

Previous studies in other organisms have classified tRFs based on the length and the region of the parent tRNA from which these fragments originate (Keam and Hutvagner 2015). We grouped small RNA reads that mapped to planarian tRNAs based on size as 18–24 and 25–35 nt. Among the two fragment size distributions, we observed 25–35 nt to be the dominant population with ∼1% to 5.8% reads mapping to tRNAs as compared to the 0.5% to 1.1% of 18–24 nt reads (Fig. 2B; Supplemental Table S3). Further, to understand the region on the tRNA from which these small RNAs arise, we plotted the per base coverage for these two sized populations over the total length of the parent tRNA. Per base coverage of the 18–24 nt species showed enrichments for two different tRNA fragment species; the tRF-5s, which originate from the 5′ end of the tRNA; and the itRFs, those that originate from the anticodon region and extend into the 3′ arm of the tRNA (Fig. 2C,D). Moreover, per base coverage for the 25–35 nt reads across the length of the tRNA suggested that these reads predominantly arise from the 5′ half of the tRNA (Fig. 2C′,D). Our analysis also revealed that among the 18–24 nt species, the itRFs are processed as a homogenous size of 20 nt, whereas tRF-5s were processed into three dominant size pools—18, 21, and 24 nt (Supplemental Fig. S3C).

Further analysis of these three different species of tRNA fragments revealed that the majority of reads for the tRF-5s, itRFs, and 5′-tsRNAs correspond to a specific set of tRNAs, an observation made in other organisms as well (Chen et al. 2016; Sharma et al. 2018; Krishna et al. 2019b). The majority of the 5′-tsRNAs were processed from tRNA pseudo-GlyGCC, contributing to ∼50% of the total 5′-tsRNA (Fig. 2E; Supplemental Table S4). While it is not general practice to include pseudo-tRNAs during the mining of tRFs, the fact that we observe a large number of reads mapping to planarian pseudo-tRNAs opens up the possibility that pseudo-tRNAs could contribute to the small RNA repertoire in other systems as well. Similarly, among the itRFs, reads mapping to tRNA UNDET-Gln23 contributed to ∼56% of the reads. However, reads were evenly distributed among the top tRNAs that generate tRF-5s (Fig. 2E). Based on the tRNA to which the reads mapped, we observed that 117 planarian tRNAs are processed into all three fragments with at least one read mapping to the parent tRNA (Supplemental Fig. S3E). Further filtering these tRNAs based on a stringent read cutoff, we identified 12 tRNAs that could be processed into all three fragments (compared to 117 without cutoffs). Twenty-five tRNAs are capable of producing both tRF-5s and the 5′-tsRNAs species while 11 tRNAs are capable of producing itRFs and 5′-tsRNAs (Supplemental Fig. S3E′). Interestingly, we observed that specific groups of tRNAs are uniquely processed into a particular species of tRNA fragment; 47 tRNAs produce only itRF species, while 41 tRNA produce tRF-5s and 3 tRNAs are processed into only the 5′-tsRNA species (Supplemental Fig. S4F′; Supplemental Table S5). It was previously reported that the abundance of tRNAs dictate the utilization of tRNA to generate tRFs (Torres et al. 2019). Notably, in planaria, some of the highest expressed tRFs were processed from specific isodecoder-tRNAs that displayed high gene copy numbers (Asp-GTC and Asn-GTT) with a corresponding low codon usage across the planarian genome (Supplemental Fig. S2B). We next validated the expression of the identified planarian tRFs using northern hybridizations and/or stem–loop RT-PCR (Fig. 2F,F′). First, we probed for the expression of the two highest expressed 5′-tsRNAs (Pseudo GlyGCC10 and UNDET-Gly_17) and observed a band corresponding to the size of the 5′-tsRNA. As a control, we probed for a tRNA (SUP-TTA_2) to which no small RNA reads mapped, and as predicted, we observed only the tRNA and no smaller fragments (Fig. 2F). Additionally, we also probed for the 3′ half of UNDET-Gly-17, and as predicted by our bioinformatic analysis, northern blots failed to pick small tRNA fragments, suggesting the absence of 3′-tsRNAs or tRF-3s for UNDET-Gly_17 (Fig. 2F). We next validated the expression of itRFs and tRF5s using the highly sensitive stem–loop RT-PCR method (Chen et al. 2005; Krishna et al. 2019b) as the expression of these small RNAs were considerably lower than those of 5′-tsRNA (Fig. 2F′). Together, our analysis of tRNA-derived fragments in planarians identifies three different species of tRFs; the smaller tRF-5s, itRFs, and the more abundant and longer 5′-tsRNAs.

#### Spatial expression patterns of tRFs across anterior-posterior (AP) axis of *S. mediterranea*

**FIGURE 3.**
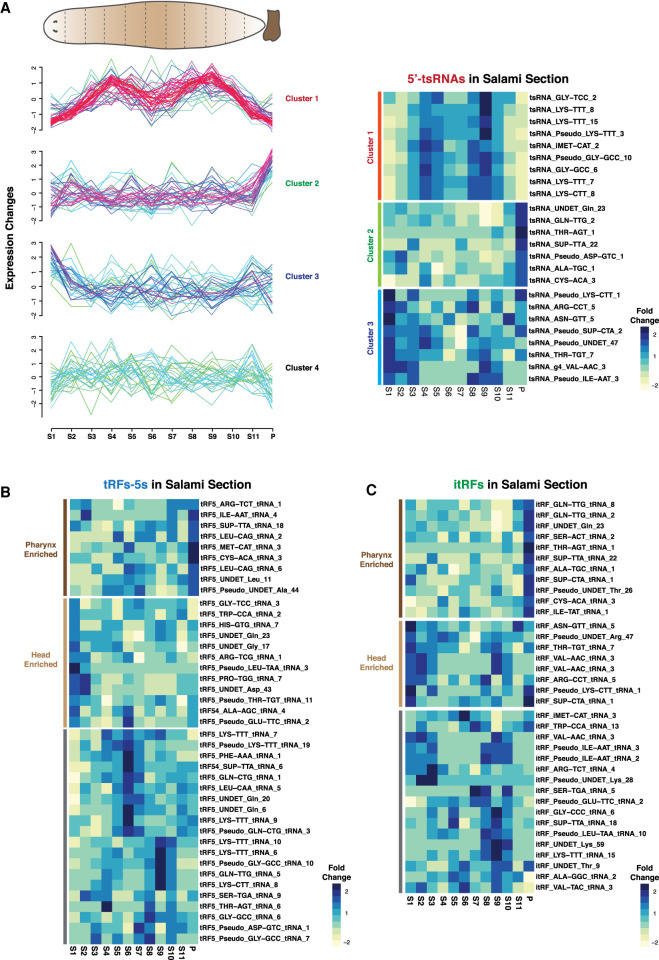
Spatial profiling of tRNA-derived small RNAs across planarian salami sections. (*A*) Expression profiles of 5′-tsRNAs across the different sections of planarian body identify three clusters of expression. In soft clustering, membership value represents how well a gene is represented in a cluster. The red and purple colors represent candidates with high membership value while yellow or green indicates candidates with low membership value. (*A*) Heatmaps of candidate 5′tsRNAs that show various patterns of observed expression. (*B*) Heatmaps of candidate tRF-5s across salami sections. The expressions are grouped based on high expression in head region, pharynx, or other regions of the planarian body. (*C*) Heatmaps of candidate itRFs across salami sections. The expressions are grouped based on high expression in head region, pharynx, or other regions of the planarian body.

We next profiled the expression of small RNAs across planarian salami sections. Initially, we analyzed the expression profiles of the most studied class of small RNAs, the miRNAs. Our analysis identified four distinct spatial clusters for miRNAs (Supplemental Fig. S4A). Consistent with previous reports, our salami section strategy showed enrichments for *miR-124*, a brain enriched-miRNA, in the anterior sections of planarians (Supplemental Fig. S4B). Subsequently, we studied the expression of the identified tRFs across the AP axis of planarians. The expression of the dominant 5′-tsRNA species could be broadly clustered into four domains. The first cluster of 5′-tsRNAs (Cluster-1), such as Gly-TCC_2, Lys-TTT_8, and Lys-TTT_15, showed enrichments in pre- and postpharyngeal regions with low expression in the head, tail, and pharynx (Fig. 3A; Supplemental Table S4). The expression of 5′-tsRNAs belonging to this cluster exhibited profiles similar to the expression of neoblast-specific transcripts, such as *smedwi-1, smedwi-2, smedwi-3, vasa*, and *bruli* (Supplemental Fig. S4C). The second cluster of 5′-tsRNAs were expressed uniformly across the different sections with higher expression in the pharynx (P). Some of the examples of pharynx enriched 5′-tsRNAs are Undet-Gln_23, Gln-TTG_2, Thr-AGT_1 etc. (Fig. 3A). 5′-tsRNAs of Cluster-3 were grouped based on their high expression in the head region (S1), suggesting that these could be expressed in the tissues of the head such as brain, eyes, etc. (Fig. 3A). Interestingly, although these tsRNAs were enriched in the head, their expression domains sometimes extended beyond the head regions, leading us to speculate that these tsRNAs may be expressed in the CNS or specific neurons. The fourth cluster of 5′-tsRNAs varied in expression across the sections with no definitive region of high expression. Similarly, clustering tRF-5s and itRFs based on expression profiles across salami sections resulted in three broad clusters like 5′-tsRNA clusters—those that are enriched in the head region, the pharynx region or in certain sections apart from these two regions (Fig. 3B,C; Supplemental Table S4). Our expression analysis of tRFs thus identifies distinct spatial expression of these small RNAs, suggesting they may be important for varied systemic functions in planarians.

#### Expression of 5′-tsRNA during planarian regeneration

**FIGURE 4.**
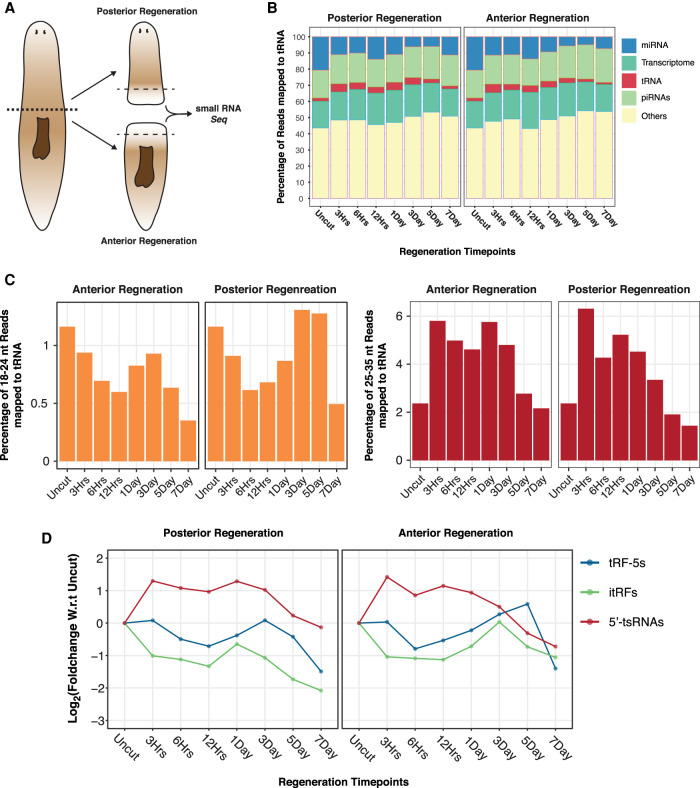
tRNA-derived fragments in planarian anterior and posterior regeneration. (*A*) Schematic describing the strategy for sequencing. (*B*) Percentage distribution of all small RNA populations across various time points of anterior and posterior regeneration. (*C*) Percentage distribution of small RNA reads mapping to tRNAs across planarian anterior and posterior regeneration. Small RNA reads were segregated into 18–24 nt and 25–35 nt. (*D*) Expression profiles of each tRNA-derived fragment population over time points of anterior and posterior regeneration shows divergent patterns.

In our previously published work on small RNAs in planarian regeneration, we identified clusters of miRNA expression in the regenerating tissue (blastema) across various time points (3, 6, 12 h, 1 d, 3 d, 5 d, and 7 d) of anterior and posterior regeneration (Fig. 4A). This data set was used to analyze the expression of different tRNA fragment species during planarian regeneration. As observed in salami sections, we obtained 2% to 5.4% of total reads (18–35) mapping to tRNAs (Fig. 4B; Supplemental Table S7). Preliminary analysis revealed dynamic changes in the different species of tRFs in both anterior and posterior regeneration. Over the course of anterior and posterior regeneration, the expression of the overall populations of 18–24 and 25–35 nt tRFs were dynamic (Fig. 4C). Similar to the size distributions of tRFs observed across salami sections, we found that 25–35 nt reads were the most abundant species during regeneration (Supplemental Fig. S5A). Per base coverage of reads that map to tRNA revealed that all the three species; itRFs, tRF-5s and 5′-tsRNAs are also expressed during regeneration (Supplemental Fig. S5B,B′). Further, the overall expression pattern of these three species revealed a divergent expression at early hours postamputation (Fig. 4D). While the collective 5′-tsRNA population doubled at 3 h postamputation, the tRF-5 species remained unchanged over this time point, and the itRFs levels decreased twofold in both paradigms of regeneration. This divergent expression for the three species of tRFs suggests distinct functionalities for these populations during regeneration.

**FIGURE 5.**
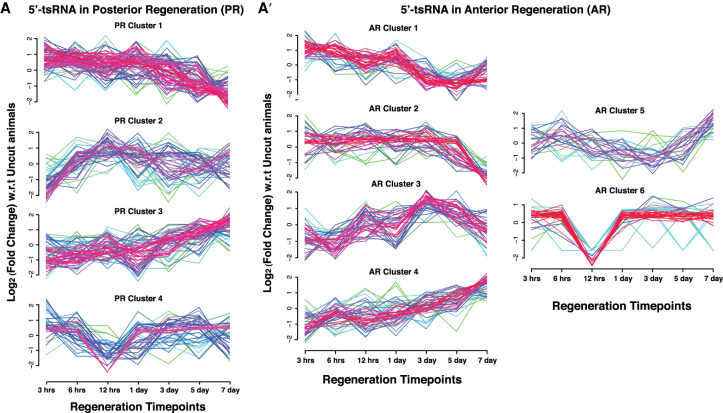
Expression profiles of 5′-tsRNAs across planarian anterior and posterior regeneration. (*A*) Expression profiles of 5′-tsRNAs across the different time points of posterior regeneration clustered into similar expression types. (*A*′) Expression profiles of 5′-tsRNAs across the different time points of anterior regenerating fragment clustered into similar expression types.

We next investigated the expression of individual 5′-tsRNAs, tRF-5s, and itRFs over the course of regeneration (Supplemental Table S8). Analysis of 5′-tsRNA expression across regenerating time points showed distinct clusters. The clusters were demarcated as anterior regeneration cluster (ARC) and posterior regeneration cluster (PRC). During posterior regeneration, our analysis revealed that 5′-tsRNAs followed four main expression profiles (Fig. 5A). PRC-1 represents the 5′-tsRNAs that are up-regulated in early time points of regeneration such as 3 hpa and their expression gradually decreases over later time points of regeneration (3 dpa–7 dpa). 5′-tsRNAs belonging to PRC-2 showed decreased expression in early time points of regeneration and peaked around 12 hpa (Fig. 5A). The expression of this cluster of 5′-tsRNAs correlates with the “second wave of wound healing response” as reported by Wenemoser et al., suggesting that these 5′-tsRNAs could be involved in the wound healing and early regeneration response. PRC-3 comprises 5′-tsRNAs that exhibit either decreased or no change in the expression until 3 dpa beyond which their expression increases (Fig. 5A). These PRC-3 5′-tsRNAs may be involved in the later stages of development and differentiation programs that set in 3 dpa during regeneration. Lastly, we identified a unique cluster (PRC-4) of 5′tsRNAs that are specifically down-regulated at 12 hpa but are comparable to uncut levels at other time points of regeneration. This cluster of 5′-tsRNAs could possibly mediate the transitions between early and late regeneration programs. We were also able to classify a unique set of 5′-tsRNAs, based on their temporal expression, as “early” (3 h–1 d) regeneration and “late” regeneration (3 d–7 d) 5′-tsRNAs (Supplemental Fig. S6A,A′).

We also examined the expression of 5′-tsRNAs in anterior regenerating animals. As compared to the posterior regeneration tsRNAs, the expression of the 5′-tsRNAs during anterior regeneration could be confidently segregated into six clusters of expression (Fig. 5A′). The ARC-1 and -2 comprises 5′-tsRNAs that have enhanced expression during early time points of regeneration (3 h onwards). The ARC-1 cluster showed a gradual decrease in the expression of 5′-tsRNAs, plummeting subsequently after day 1 postamputation (Fig. 5A′). ARC-2 5′-tsRNAs have a more prolonged period of elevated expression with a sharp decrease in expression at 7 dpa. The trends exhibited by ARC-1 and ARC-2 classes of 5′-tsRNAs would suggest a role for these small RNAs in regulating the early events of regeneration. ARC-3 and ARC-4 displayed similar expression profiles, both showing increased expression patterns at later time points of regeneration. Both these clusters showed decreased 5′-tsRNA expression in early time points of regeneration with a gradual or a sharp increase in expression around 1–3 dpa (Fig. 5A′). While ARC-3 includes 5′-tsRNAs that peak in expression around 3–5 dpa followed by a decrease at 7 dpa, the ARC-4 showed highest expression at 7 dpa, similar to the expression of the PRC-3. We speculate that ARC-3 clusters could represent the wave of 5′-tsRNAs that are essential for the formation of the head structures while the ARC-4 cluster could represent the pool that could be involved in homeostasis, growth, and organization of the head structures. ARC-5 displayed increased expression at early (3 and 6 hpa) and late (7 dpa); however, they displayed decreased expression at 3–5 dpa (Fig. 5A′). Here, we speculate that ARC-3, ARC-4, and ARC-5 could be the 5′-tsRNAs that are involved in later stages of head regeneration. We also identified a set of 5′-tsRNAs (ARC-6) that showed a sharp reduction in expression at the 12 h time point akin to the PRC-4 (Fig. 5A′).

**FIGURE 6.**
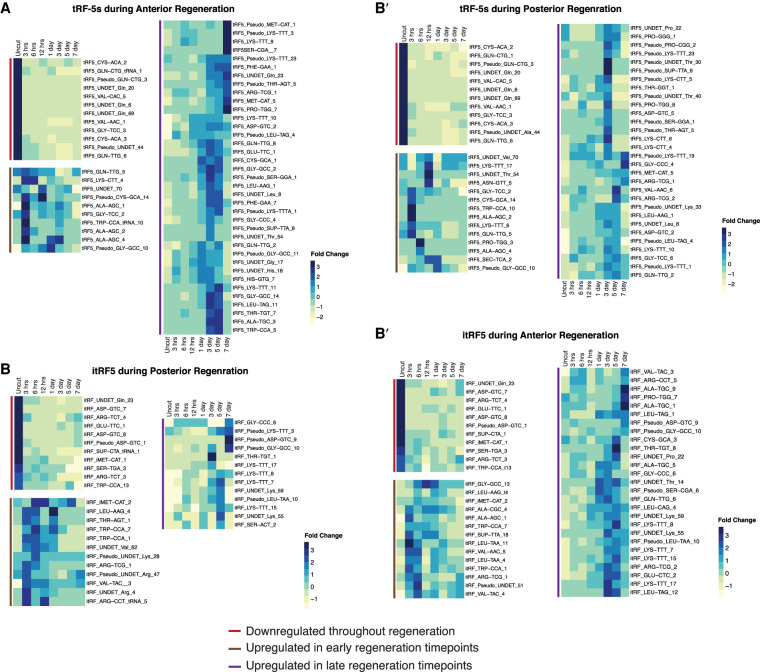
Expression profiles of tRF-5s and itRFs across planarian anterior and posterior regeneration. (*A*,*A*′) Heat maps of candidate tRF-5s during anterior and posterior regeneration. We identified three groups of expressions. tRF-5s that are down-regulated over all regeneration time points, tRF-5s that are up-regulated in early timpoints of regeneration (3rs–1 d), and late time points of regeneration (3–7 d). (*B*,*B*′) Heat maps of candidate itRFs during anterior and posterior regeneration. We identified three groups of expressions. itRFs that are down-regulated over all regeneration time points, itRFs that are up-regulated in early time points of regeneration (3rs–1 d) and late time points of regeneration (3–7 d).

Next, we profiled the expression patterns of tRF-5s and itRFs at different time points of anterior and posterior regeneration. Similar to the expression patterns observed with 5′-tsRNAs, we were able to categorize the expression of tRF-5s and itRFs into early and late waves of expression (Fig. 6A,A′,B,B′; Supplemental Table S8). Our analysis also revealed a group of 5′-tsRNAs potentially not involved in the regeneration process as evidenced by the down-regulation of these small RNA over all the tested regeneration time points (Figs. 5A′, 6A,B,B′). To distinguish the tRFs that are specific to either anterior or posterior regeneration, and those that are involved in a common regenerative program (irrespective of anterior or posterior regeneration), we analyzed the overlapping tRFs between these two paradigms. Interestingly, during the early regeneration response, a greater percentage of tRFs were common between anterior and posterior regeneration (around 25% for itRFs, 50% for tRF-5s and 45% for 5′-tsRNAs) (Supplemental Fig. S6B). However, during the late response, a large percentage of tRFs emerged specific to head regeneration, possibly those that are important for the regeneration of the head structures. We also observed a concomitant decrease in the common tRFs (for tRF-5s and 5′-tsRNAs) and tRFs specific to the tail regeneration (Supplemental Fig. S6B). Collectively, our analysis identified dynamic expressions of several tRFs throughout planarian regeneration.

#### Planarian 5′-tsRNAs interact with all three SMEDWIs

**FIGURE 7.**
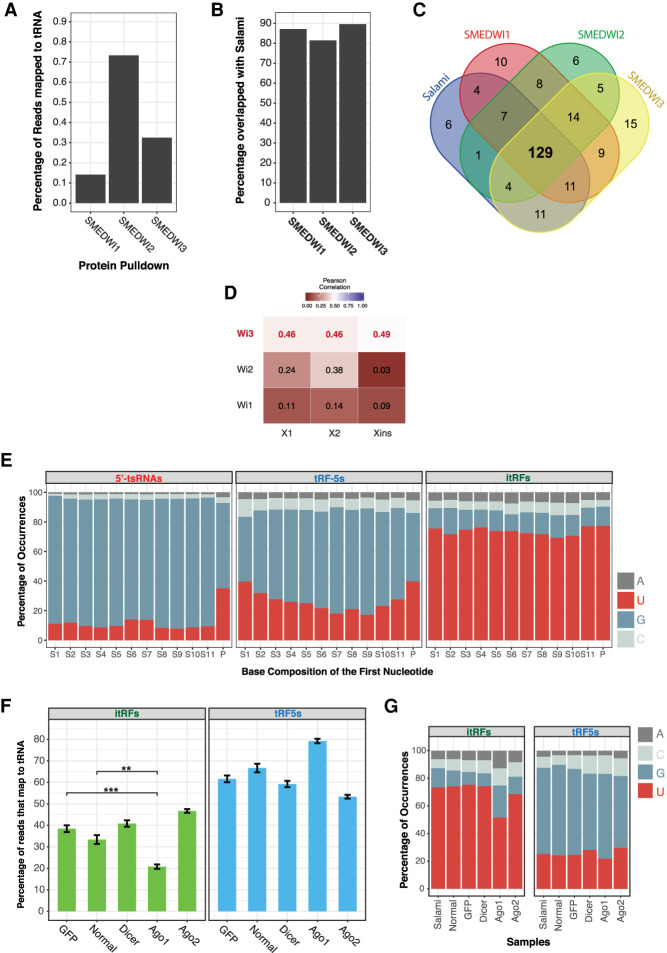
5′-tsRNA interaction with PIWI and sequence signatures in tRNA-derived fragments. (*A*) Percentage of SMEDWI-1, -2, and -3 interacting RNAs that map to tRNAs. (*B*) Percentage of identified planarian 5′tsRNAs associating with SMEDWI proteins in planaria. (*C*) Pie chart of the common 5′-tsRNAs identified by salami sections and the three SMEDWI-associating 5′-tsRNAs. (*D*) Correlation plot of 18–35 nt small RNA reads mapping to tRNA across SMEDWI-1, -2, and -3 with 18–35 small RNA reads mapping to tRNAs in X1, X2, and Xins cell populations. (*E*) Base preference of 5′tsRNAs, tRF-5s, and itRFs at the first position, across planaria body sections. itRFs exhibit a strong enrichment for “U.” (*F*) Changes in the itRF and tRF-5 population upon *Dicer*, *Ago1*, and *Ago2* knockdown show decreased itRF population in *Ago1* KD conditions compared to the controls (*GFP* dsRNA fed animals and untreated animals). Error bars represent S.E.M. (*G*) Base preference of itRFs at the first position across the three knockdown conditions (*Dicer*, *Ago1*, and *Ago2*). (**) *P*-value <0.01; (***) *P*-value <0.001.

Several studies have identified potential enzymes and factors responsible for processing of tRFs (Yamasaki et al. 2009; Tuorto et al. 2012; Guzzi et al. 2018). Of the different species of tRFs, processing of tRNAs by angiogenin to produce tsRNAs (or tiRNAs or tRNA halves) under stress conditions has been the most studied (Yamasaki et al. 2009; Su et al. 2019a). Sequence homology-based surveys suggested that *S. mediterranea* lacks proteins homologous to angiogenin. This implied that the tsRNAs in planarians are processed by a completely different mechanism. Recent evidence in mammalian systems have also arrived at similar conclusions that 5′-tsRNAs could be processed in an angiogenin-independent manner (Krishna et al. 2019b; Su et al. 2019b). Another protein that is abundantly expressed in planarians and one that has been reported to associate with 5′-tsRNAs in other systems is Piwi (Keam et al. 2014). Planarians express three piwi proteins SMEDWI-1, SMEDWI-2, and SMEDWI-3 (Reddien et al. 2005; Palakodeti et al. 2008; Kim et al. 2019). A recent study in planarians identified the RNAs associated with the three SMEDWI proteins to understand their function (Kim et al. 2019). We mapped this data set to our annotated tRNAs to explore if 5′-tsRNAs associate with these proteins. The reads that mapped tRNAs were predominantly of the size 30–35 nt suggesting that SMEDWIs interact with tsRNAs (Supplemental Fig. S7A). Our analysis revealed that a small subset of PIWI-interacting RNAs map to tRNAs with SMEDWI-2 showing the highest association (Fig. 7A; Supplemental Table S9). It is not surprising that tsRNAs represent only a minor fraction of PIWI-interacting RNAs considering the fact that tsRNAs make up a small fraction of the total small RNAs in planarians, and with piRNAs being the primary interactors of PIWI proteins. However, it is important to note that 80%–90% of all the planarian 5′-tsRNAs associate with the SMEDWI proteins indicating PIWIs to be essential for the biogenesis and/or functioning of 5′-tsRNAs in planarians (Fig. 7B). Our analysis also revealed that all SMEDWI proteins in planarians associate with a large pool of common 5′-tsRNAs (Fig. 7C). This observation suggests two possibilities, that 5′-tsRNAs use different routes of biogenesis/function through PIWI proteins, and planarian 5′-tsRNAs may interact differently with these three proteins across the three planarian cell populations. To explore the latter, we investigated if there is any correlation between the 5′-tsRNAs that associate with the three PIWI proteins and the tsRNAs expressed in the three different cell populations in planarians (X1, neoblasts; X2, largely comprising of progenitors; and Xins, the differentiated cells). To perform this analysis, we used our previously published small RNA data from X1, X2, and Xins cell populations (Sasidharan et al. 2013a). Our analysis revealed that 5′-tsRNAs associated with SMEDWI-3 showed the highest level of correlation with these three cell types (Fig. 7D; Supplemental Table S10). It has been previously reported that SMEDWI-3 is also expressed in differentiated cells, suggesting the reason for stronger correlation with X2 and Xins populations compared to SMEDWI-1 and 2 (Shibata et al. 2016; Kim et al. 2019). SMEDWI-1 associating 5′-tsRNAs correlated the least with the three cell types. In conclusion, our data suggests that SMEDWI proteins could be critical for planarian tsRNA biogenesis and function.

#### AGO1-based processing of itRFs in *S. mediterranea*

Understanding sequence features and base compositions have helped identify and characterize small RNAs across various systems (Wang 2013). Sequence analyses of tRFs have provided valuable insights into the processing and functionality of tRFs. A “GG” dinucleotide that is present across a majority of tRNAs and thus retained in the tsRNAs have been shown to be important for the repressive role of tsRNAs in translation (Sobala and Hutvagner 2013). Similar sequence analysis of 5′-tRFs identified TOG motifs (terminal oligoguanine) that facilitate G-quadruplex based interactions with YBX1 (Lyons et al. 2017). Interestingly, analysis of planarian tRF base compositions revealed strong signatures for “U” at the first base of itRFs (Fig. 7E; Supplemental Fig. S7B). Similar preference for “U” among itRFs was observed across all the data sets used in this study (Supplemental Fig. S7C). This enrichment of “U” at the first position was absent in tRF-5s and 5′-tsRNA that preferred a “G” (Fig. 7E; Supplemental Fig. S7B). It is possible that this enrichment of “U” could potentially arise because the parent tRNA might have an enrichment for “U” at that particular position. To test this hypothesis, we first checked the start positions of itRFs on the tRNA and found that the majority of the reads originated from the 34th position on the tRNA (Supplemental Fig. S7D). We next examined if all the tRNAs have an enrichment for “U” at this (34th) position. Our analysis revealed that there was a preference (∼40%) for U at the 34th position of the tRNA (Supplemental Fig. S7E). In comparison, we observed a higher degree of enrichment (∼70%) at the first position of all the itRFs (Fig. 7E), suggesting a selective processing that results in 5′ U enrichment. Interestingly, although positions 33 and 35 on tRNAs exhibited similar base preferences for “U,” only a very few itRFs were processed from these positions (Supplemental Fig. S7D,E).

The observed enrichment of “U” at the 5′ end is characteristic of several siRNA/miRNA that are processed by DICER and/or associate with AGO (Mi et al. 2008; Cheloufi et al. 2010; Ghildiyal et al. 2010; Hoehener et al. 2018). Moreover, the itRFs also display a more homogenous size distribution of ∼20 nt that additionally suggests that itRFs may be processed by DICER or AGO (Supplemental Fig. S3C). Planarians express two Ago genes; *Ago1* and *Ago2*, with *Ago2* knockdown (KD) showing severe phenotypes (Li et al. 2011). To understand the processing of itRFs in planarians, we knocked down *Dicer*, *Ago1*, and *Ago2*. Similar to the phenotypes observed previously (Li et al. 2011), all of the *Dicer* and *Ago2* KD animals lysed, while very few *Ago1* KD animals (20%) showed lethal phenotypes (Supplemental Fig. S8A). We performed small RNA sequencing from these animals to identify changes in the 18–24 nt tRF pools. As expected, we observed a decrease in miRNAs among the *Dicer* KD animals compared to the control animals (*GFP*: animals fed with *GFP* dsRNA; and Normal: animals not administered dsRNA) (Supplemental Fig. S8B), but surprisingly, we observed no significant changes in tRF-5 or itRF populations (Fig. 7F). Similarly, we observed no significant changes in miRNA, tRF-5, or itRF levels in *Ago2* KD animals (Fig. 7F; Supplemental Fig. S8B). This suggested that DICER and AGO2 may not be involved in the processing of itRFs. However, we interestingly observed an approximately twofold decrease in the itRF population in the *Ago1* KD animals, while the miRNA and the tRF-5 population remained largely unchanged (Fig. 7F; Supplemental Fig. S8B,C). Concurrently, knockdown of *Ago1* in planarians resulted in a decrease in the preference for 5′ U among the itRFs (Fig. 7G). It has been previously shown that Argonauts generate small RNAs in a dicer-independent mechanism (Diederichs and Haber 2007; Cheloufi et al. 2010; Kretov et al. 2020), and our data suggests that AGO1 in planarians may be involved in the dicer-independent processing and/or maintaining the stability of the itRFs.

## DISCUSSION

tRNA-derived fragments have been implicated in regulating various cellular processes through diverse mechanisms (Keam and Hutvagner 2015). However, most studies aimed at understanding tRFs have been confined to a particular cellular process. Our study for the first time identifies and suggests a possible role for these small RNAs in regulating a complex biological process that is an embodiment of several cellular and molecular events, such as planarian regeneration.

Due to a lack of a comprehensive tRNA annotation in planarians, devising a stringent tRNA annotation pipeline was imperative to identify tRFs in planarians. Our pipeline identified 457 tRNA genes across the *Schmidtea mediterranea* genome. These 457 tRNA genes can be broadly categorized into two groups: (i) standard tRNAs that code for 20 standard amino acids (347); and (ii) pseudo tRNAs (110). Interestingly, our prediction of tRNAs in planarians failed to identify tRNAs for seven anticodons. This is, however, a common occurrence as several genomes use near-cognate tRNAs to compensate in the absence of the correct codon anticodon pair through wobble base-pairing (Chan and Lowe 2009). Alternatively, the seven unidentified anticodons could belong to the UNDET category of tRNAs. Surprisingly, one of the tRNAs exhibiting high gene copy number was a suppressor tRNA, SUP-TTA tRNA with 17 genes (spread across 52 genomic loci) in the planarian genome (Fig. 1D). Suppressor tRNAs are mutants of standard tRNA genes that recognize a stop codon, and thus deliver an amino acid to these positions instead of termination (Eggertsson and Soll 1988; Hatfield et al. 1990). The large number of suppressor tRNAs in planarians evokes an exciting possibility that these tRNAs could result in increased protein lengths, thus altering their function. Understanding the function of these SUP-tRNAs roles in translation regulation will add an additional layer of gene regulation in planarian biology. Our analysis also revealed a positive correlation between tRNA gene copy number and codon usage in planarians. Understanding the tRNA availability and codon has been important in shaping the translational landscape across organisms (Varenne et al. 1984; Sørensen et al. 1989; Yu et al. 2015). The selection of the “optimal” codons will be critical for optimizing heterologous expression of genes in the development of planarian transgenics.

Characterization of the tRNA-derived fragments in planarians revealed three main classes of tRFs, the 18–24 nt species comprised of two species—the tRF-5s, itRFs, and the 25–35 nt 5′ tsRNAs. As observed in other systems (Peng et al. 2012; Chen et al. 2016; Sharma et al. 2016a; Jehn et al. 2020), 5′-tsRNAs were the dominant tRF species in planarians. Notably, reads mapping to the 3′ end of planarian tRNAs were insignificant, which was further validated by the absence of smaller tRNA fragments in northern hybridization. This suggests an active role for the stable 5′- tsRNAs, while its 3′ counterparts could be actively cleared from the system. However, we cannot rule out the existence of 3′-tRNAs from other tRNAs as 3′ halves may carry modifications and/or be charged with amino acids that may require additional treatments to make them susceptible to be detected by high-throughput sequencing protocols.

Expression analysis of the observed three species of tRFs across the planarian sections revealed four main clusters showing distinct spatial expression. A subset of 5′tsRNAs showed expression patterns similar to neoblast-enriched transcripts, suggesting that these 5′-tsRNAs could be key players in maintaining the stem cells’ homeostasis and differentiation. Further, our analysis also revealed that certain 5′-tsRNAs, itRFs, and tRF5s are enriched in the head and pharyngeal region, while some are expressed throughout the planarian body. Our spatial expression analysis of tRFs adds to the catalog of posttranscriptional regulatory mechanisms that exist in planarians. Analysis of tRFs during planarian regeneration revealed a divergent expression for all three species, suggesting that these three species could use different modes of regulation during regeneration. It has been observed that 5′-tsRNAs largely influence the translation of transcripts, while the shorter 5′-tRFs and itRF may act akin to miRNAs, similar to what has been observed in *Drosophila* tRFs (Kumar et al. 2014; Karaiskos et al. 2015; Kuscu et al. 2018; Luo et al. 2018). It would be interesting to understand the regulatory mechanisms these small RNAs use to orchestrate planarian regeneration. Inspection of individual tsRNA expression revealed a dynamic change in expression during anterior regeneration (six clusters) as compared to posterior regeneration (four clusters). This observation could possibly be explained by the need to regenerate and reorganize the complex structures that make up the head as compared to the structures of the tail region. Our analysis also facilitated the dissection of “early” and “late” response tRFs. The “early” tRFs could be essential for the wound closure and wound healing program, whereas the “late” tRFs could possibly regulate the differentiation and remodeling.

Our study also sheds light on the biogenesis and/or the modes of action of these small RNAs. Analysis of the parent tRNAs from which planarian tRFs are processed revealed that a majority of these small RNAs are processed from specific sets of tRNA. Similar to higher organisms, in planarians we observed that Gly, Gln, and Asp were the most predominantly processed tRNAs. It is interesting to note that this selective processing of tRNAs is evolutionarily conserved while our understanding of this process remains poor. 5′-tsRNAs (or tiRNAs or tRNA halves) have been shown to be produced by the endonucleolytic cleavage of tRNAs by angiogenin (Yamasaki et al. 2009). Cleavage by angiogenin results in the tsRNAs carrying 2′–3′ cyclic phosphates that are not amenable for conventional sequencing protocols (Honda et al. 2015). However, the data used in this study were obtained using conventional sequencing protocols. Moreover, we failed to identify a homolog for angiogenin in planarians. This strengthens our reasoning to believe that 5′-tsRNAs in planarians are processed in an angiogenin-independent manner. However, studies in humans have suggested that tsRNAs could potentially be bound by PIWI (Keam et al. 2014). Our analysis of the recently published SMEDWI-1, -2, and -3 ClIP-seq data suggested that the majority of 5′ tsRNAs interact with PIWI proteins in planarians. It is interesting to note that SMEDWI-3, a piwi protein implicated to target coding transcripts, interacts with 5′ tsRNAs in all three cell compartments (X1, X2, and Xins). This evokes the possibility of PIWI targeting some of the coding transcripts identified in a previous study, mediated by the 5′-tsRNAs (Kim et al. 2019). Lastly, we observed a high base preference for “U” among the itRFs, suggestive of miRNA-like processing. Canonically, miRNAs and siRNAs are processed by DICER with a preference for “U” at the 5′ end (Hoehener et al. 2018). However, knockdown of *Dicer* in planarians failed to show any differences in the itRF population. Interestingly, tRF-5s that were implicated to undergo Dicer-based cleavage in other systems (Cole et al. 2009; Martinez et al. 2017) also remained unaltered in *Dicer* KD, suggesting that tRFs in planarians are DICER-independent. However, in several systems, Argonauts have been shown to process small RNAs in a dicer-independent pathway (Diederichs and Haber 2007; Cheloufi et al. 2010; Kretov et al. 2020). Planarian AGO2 has been suggested to be critical for miRNA-based regulation (Li et al. 2011), while the function of AGO1 remains unexplored. Knockdown of *Ago1* in planarians specifically resulted in an approximately twofold decrease in itRF population, while this population remained unchanged in *Ago2* KD conditions. These results indicate a novel role for AGO1 in the maintenance of the itRF population in planarians. It is also noteworthy that similar associations of AGO1 with 5′-U enriched small RNAs have been observed in *Drosophila* and *Arabidopsis*, suggesting that planarian AGO1 may function through similar mechanisms (Mi et al. 2008; Ghildiyal et al. 2010).

In conclusion, we present the first ever report and characterization of planarian tRFs using high-quality tRNA annotations. The regulatory roles of tRFs remain poorly understood at an organismal level, and our characterization of these small RNAs in planarians solidifies the notion that tRFs are an important family of small RNAs with impactful regulatory roles across all life forms. Further, the dynamic expression of planarian tRFs during regeneration suggests active roles for these small RNAs in complicated biological processes. Considering the versatility in tRF-mediated gene regulation in other systems, our study opens a new avenue of research in the quest to understand the process regeneration. Moreover, molecular tractability of planarians makes them an ideal invertebrate model system to explore the function and biogenesis of these small RNAs in vivo.

## MATERIALS AND METHODS

### Predicting putative tRNA gene families

We used dd_Smes_G4 (Grohme et al. 2018) assembly of the *Schmidtea mediterranea* genome to identify putative tRNA genes. We used two programs to identify tRNA gene loci across the planarian genome: tRNA-SCAN-SE (version 1.3.1) and Aragorn (Lowe and Eddy 1997; Laslett and Canback 2004). We enabled intron prediction for both the programs and used the following parameters to predict: tRNA-SCAN_SE (*-G -y -l -o -f -m -p*) and Aragorn (*-t -gcstd -I -seq -br -fasta -o*). Both the programs predicted ∼4100 sites across the planarian genome (4115—tRNA-SCAN-SE and 4143—Aragorn). We clustered the sequences predicted by both the programs using CD-HIT with 90% sequence similarity cutoff and identified 708 unique sequences (Li and Godzik 2006a). Among 708 sequences, 302 were predicted by both the programs; the remaining 406 sequences (132 and 274) were predicted only by tRNAScan-SE and Aragorn, respectively. We further removed false positives from these 708 predicted sequences using tRNA-SCAN-SE (version 2.0.5, *-o -f -m -a -l -p –detail -y –isospecific –thread*) (Chan and Lowe 2019). This improved version of the algorithm is known to have incorporated methodologies with improved probabilistic search and gene models. We finally narrowed down 457 unique sequences which could code putatively for tRNA genes. We used VARNAv3-93 for obtaining secondary structures of tRNA (Darty et al. 2009) as shown in Figure 1E.

We further used scores derived from TFAM 1.0 classifier (*-E -t -s*) to assign an amino acid to the UNDET tRNAs identified from our method (Tåquist et al. 2007). Using TFAM classifier, we identified three initiator tRNA (*iMet*) in the planaria genome (Fig. 1; Supplemental Table S1). iMet tRNA was identified based on sequence features highlighted in Figure 1E and the isotype specific score from tRNA-SCAN-SE (v2.0.5). We also aligned predicted planarian tRNA sequences codon-wise using MacVector and given as extended Supplemental Figure 1.

### Codon usage calculation

To calculate codon usage in planaria, we downloaded the latest version of transcriptome annotation (SMESG—repeat filtered) that has 30,917 genes (∼59,800 isoforms) from planmine (Rozanski et al. 2019). We only considered ORFs which are of length ≥100 and ≤10,000 nt. We also removed the gene sequence which has Ns in the ORF regions. Codon usage is the measure of frequency of occurrence of all the possible three letter codons in coding transcripts (ORFs). We wrote a custom made perl script to calculate this parameter.

#### Frequency per 1000 codons

This index is a ratio of occurrence of each codon to the total number of triplets from all coding ORFs and normalized per 1000 codons. Frequency per 1000 codons gives the global profile of codon usage for a particular organism (Supplemental Table S2).

$$\mathrm{Frequency}=\frac{\mathrm{Occurrence}\;\mathrm{of}\;a\;\mathrm{particular}\;\mathrm{codon}}{\mathrm{Total}\;\mathrm{number}\;\mathrm{of}\;\mathrm{codons}\;\mathrm{across}\;\mathrm{all}\;\mathrm{transcripts}}\times 1000$$

Percentage of tRNA loci per codon is calculated similar to codon fraction for each codon.

### Small RNA data sets in planaria

We downloaded the publicly available small RNA data sets of planaria from NCBI-SRA. Planarian cell population (X1, X2, and Xins) and regeneration time point data were downloaded from SRA065477 (Sasidharan et al. 2013b). Smed-piwi pulldown data was downloaded from GSE122199 (Kim et al. 2019).

### Identification of tRNA-derived fragments in planaria

We used dd_Smes_G4 (Grohme et al. 2018) assembly of the *Schmidtea mediterranea* genome and tRNAs predicted from this study for analysis. Small RNA sequencing data downloaded from SRA were converted to fastq files using the SRA-toolkit (https://ncbi.github.io/sra-tools/). From the sequencing reads, we trimmed TruSeq small RNA adapters using the Cutadapt program (-f fasta -b TGGAATTCTCGGGTGCCAAGG -O 5 -m 6 -o) (Martin 2011). Adapter trimmed reads were aligned to flatworm (planarian) rRNA sequences retrieved from NCBI and unmapped reads were used for further analysis. We downloaded planarian miRNA sequences from miRbase (Griffiths-Jones et al. 2008) and piRNA sequence coordinates from Kim et al. For analysis, we considered reads ranging from 18 and 35 nt and mapped these to the genome and other databases using bowtie v1.1.2 (*-f -v 2 -p 20 –un*) (Langmead et al. 2009). We used two mismatches as a constant parameter for mapping all the reads used in this study, as we did not observe much deviation in our results with varying mismatches ranging from 0 to 2. To calculate the percentage tsRNA reads that mapped to the genome, we calculated the ratio of reads of a particular size that mapped to tRNA to the total reads that mapped to the genome. Further, we calculated per base tRNA coverage using the following formula given below. We used the coverage values obtained to plot the tRNA coverage heatmaps and area plots. We used customized perl script for all the analysis used in this study. We used the R ggplot2 library for plotting (Ginestet 2011). We followed a similar analysis pipeline as described in Krishna et al. (2019b).

$$\begin{array}{rl} \mathrm{Perbase}\;\mathrm{coverage}= & \frac{\mathrm{Number}\;\mathrm{of}\;\mathrm{reads}\;\mathrm{aligned}\;\mathrm{to}\;\mathrm{particular}\;\mathrm{base}\;\mathrm{of}\;\mathrm{tRNA}}{\mathrm{Total}\;\mathrm{number}\;\mathrm{of}\;\mathrm{reads}\;\mathrm{mapped}\;\mathrm{to}\;\mathrm{all}\;\mathrm{the}\;\mathrm{tRNA}\;\mathrm{in}\;\mathrm{that}\;\mathrm{sample}} \end{array}$$

### Small RNA counts and data normalization

We obtained raw read counts mapping to individual tRNA using customized perl script. We used the well-established DESeq algorithm for normalization of the sequencing data and identification of differentially expressed tRFs (adj. *P*-value <0.05) (Anders and Huber 2010). All the statistical tests were done in R (R Development Core Team 2016). The normalized values obtained from DESeq is used for clustering the tsRNAs based on expression across multiple data sets. Top 10 candidates were represented as percentages and plotted in a pie-chart as shown in Figure 2D. We used MFuzz (R package), and soft clustered expression values across multiple time points to obtain dominant cluster patterns (Kumar and Futschik 2007). All the heatmaps depicting expression changes are plotted using a R package pheatmap, (https://cran.r-project.org/web/packages/pheatmap/index.html).

### Small RNA sequencing from salami sections

Asexual planarians were killed and sliced into 12 sections as described in Stückemann et al. (2017). The sliced pieces were put in TRIzol to isolate RNA. Small RNA libraries were made using Illumina TruSeq Small RNA Prep kit and later sequenced on Nextseq500 machine. We analyzed the data as described above in the methods. We averaged the normalized value derived from each fragment to get an estimate of expression in whole animal.

### *Smedwi* pulldown data correlation

We downloaded smed-PIWI protein pulldown data from GSE122199 and processed it to identify putative tRFs as described in the methods above. We normalized the individual tRFs using DESeq. To draw correlations between piwi-associating tRFs and planarian cell populations, we used small RNA sequencing data from X1, X2, and Xins populations from SRA065477. Small RNA sequencing data was processed as described above in methods and normalized values from three distinct cell populations are correlated with the pulldown data (Supplemental Table S10). We calculated the Pearson correlation and plotted it as a matrix using R and ggplot2 module.

### Base preference in small RNA reads

We categorized reads mapping to the three identified pools (tRF5s, itRFs, tsRNAs) based on read length and position in which reads align to an tRNA. We then size segregated reads which fall under these categories and calculated per-base preference using custom perl script. Per base coverage is calculated as occurrence of A,T,G,C at each position divided by total number of reads under each category. The derived percentages were later plotted using R ggplot2 package.

### Understanding dynamic processing of tRNAs

From the sequencing data, we observed three distinct pools of tRNA-derived small RNA fragments in planaria. To check if there are any overlaps in tRNAs that are processed in generating these fragments (tRF-5s, itRFs, and tsRNAs), we devised this following strategy. To assign if a particular tRNA is giving rise to one specific pool, it must satisfy these three criteria: (i) >90% of reads mapping to that particular tRNA should map either before (5′ end) or after the (3′ end) anticodon position. This is relative to the fragments that are assessed. For example, if the fragment is itRFs, >90% of reads mapping to that particular tRNA should map after the anti-codon (3′end); (ii) the remaining 10% reads should be less than 10 reads, as it would be a negligible amount to define it as one of the pools; and (iii) since the three identified species are of varied lengths (tRF5s, itRFs—18–24 nt and tsRNAs—25–35 nt), we made sure that the expression of the third species is less than the median normalized value and has fourfold lesser reads mapping. For example, if the fragment is itRFs (18–24 nt of length), at least fourfold lesser number of reads (w.r.t 18–24 nt reads mapping at 3′end) of length 25–35 nt should map to the 5′ end of tRNA. The number of 25–35 nt reads mapping at the 5′ end should be less than the median normalized value. Based on these criteria, we classified tRNAs into capable of coding all three or either of two or specific to one pool of tRNA derived small RNA fragments. The number of tRNAs that are categorized is shown in Supplemental Figure S3E.

### Northern hybridizations

The RNA blot was performed as described previously (Shivaprasad et al. 2012; Tirumalai et al. 2020). An amount of 10 µg of total RNA was isolated from whole planaria (homeostasis) and resuspended in 8 µL loading buffer (0.10% bromophenol blue, 0.10% xylene cyanol in 100% de-ionized DEPC-treated formamide), heated at 95°C for 1 min, and loaded on to a 15% denaturing polyacrylamide gel (a 19:1 ratio of acrylamide to bisacrylamide, 8 M urea). The gel was run at 100 V for 3 h and then transferred to a Hybond N+ membrane by electroblotting at 10 V overnight at 4°C. After UV crosslinking (UVP), hybridization was performed at 35°C for 12 h in UltraHyb-Oligo buffer (Ambion) containing desired probes (*Pseudo_GlyGCC_10* – TACCACTGAACCACCAATGC; *UNDET-Gly_17* – TACCACTGAACCACCGATGC; *UNDET-Gln_23* – ACGCCTACACCATGGACCTC; *GlyTCC_6* – GACCGTTACACCACAATCGC; *Asn-GTT_5* – AATTGCGCCACGGAGGCTC; *iMet-CAT_1* – TCCACTGCGCCACTCTGCT; *SUP-TTA_2* – CCGCTTACACCATCGAACC). DNA oligos complementary to candidate 5′-tsRNAs, tRF-5s, itRFs were end-labeled with ^32^P-ATP (Board of Radiation and Isotope Technology) using polynucleotide kinase (NEB), purified through MicroSpin G-25 Columns (GE Healthcare), and were used as probes. The blot was washed twice with 2× SSC, 0.5% SDS for 30 min at 35°C. The signal was detected after exposure on a phosphorimager screen using a Molecular Imager (GE Healthcare). All the tRF candidates were analyzed in five biological replicates.

### Stem–loop RT-PCR validation of tRFs

Stem–loop RT-PCR based validations of tRFs were performed as described previously (Krishna et al. 2013, 2019b). A total of 1 µg of planarian RNA was reverse transcribed with 10 µM of stem–loop carrying reverse transcription primer designed specifically to individual tRF using SuperScript III (18080093, ThermoFisher). The reverse transcribed product was amplified in a PCR reaction using 2.5 µM tRF-specific forward primer and a universal reverse primer. The amplicons were run on a 10% polyacrylamide gel. The size of the amplicon for a 20 nt tRF would be 70 bp and for a 30nt tRF would be 80 bp. The primer sequences used are tabled in Supplemental Table S12.

### Planarian culture and RNAi experiments

Animals used in this study belong to the sexual strain of species *Schmidtea mediterranea*. They were maintained at 20°C in planarian media (2 mM NaCl, 0.1 mM KCl, 0.1 mM MgSO_4_, 0.12 mM NaHCO_3_ in distilled water) and fed beef liver paste. Animals were starved 2 wk prior to experiments. Animals used in this study belong to the sexual strain of species *Schmidtea mediterranea*. They were maintained at 20°C in planarian media (2 mM NaCl, 0.1 mM KCl, 0.1 mM MgSO_4_, 0.12 mM NaHCO_3_ in distilled water) and fed beef liver paste. Animals were starved 1 wk prior to any experiments. For knockdown, homeostatic animals were fed beef liver paste with in vitro synthesized dsRNA (synthesized using T7-flanked amplicons) for *GFP*, *Dicer1(dd_6814)*, *Ago1(dd_7594)*, and *Ago2(dd_3861)* genes. A total of 3–4 mg/mL concentration of dsRNA was mixed with beef liver paste in a ratio of 50:15 (beef :dsRNA) mL. The animals were fed dsRNA-beef mixture every alternate day for seven feeds. Animals were added to TRIzol once phenotypes were observed. RNAi was performed in biological duplicates with *N* = 10 for each replicate. Small RNA libraries were made using Illumina TruSeq Small RNA Prep kit and later sequenced on Nextseq500 machine. For all the RNAi conditions, we sequenced both biological and technical replicates. As an additional control for our analysis, we performed sequencing from animals that were not administered dsRNA (referred to as Normal animals). We analyzed the data as described above in the methods. T7 Primers used to synthesize dsRNA are given in Supplemental Table S12.

## DATA DEPOSITION

The small RNA sequencing data from planarian salami sections generated as a part of this study are deposited in NCBI-SRA under the project id SRP277000 (PRJNA646861).

## SUPPLEMENTAL MATERIAL

Supplemental material is available for this article.

## Supplementary Material

Supplemental Material

## References

[RNA077701LAKC1] Anders S, Huber W. 2010. Differential expression analysis for sequence count data. Genome Biol 11: R106. 10.1186/gb-2010-11-10-r106 20979621 PMC3218662

[RNA077701LAKC2] Bansal D, Kulkarni J, Nadahalli K, Lakshmanan V, Krishna S, Sasidharan V, Geo J, Dilipkumar S, Pasricha R, Gulyani A, 2017. Cytoplasmic poly (A) binding protein (PABPC2) critically regulates epidermal maintenance and turnover in planarian *Schmidtea mediterranea*. Development 144: 3066–3079. 10.1242/dev.152942 28807897 PMC5611960

[RNA077701LAKC3] Bazzini AA, Viso F, Moreno-Mateos MA, Johnstone TG, Vejnar CE, Qin Y, Yao J, Khokha MK, Giraldez AJ. 2016. Codon identity regulates mRNA stability and translation efficiency during the maternal-to-zygotic transition. EMBO J 35: 2087–2103. 10.15252/embj.201694699 27436874 PMC5048347

[RNA077701LAKC4] Chan PP, Lowe TM. 2009. GtRNAdb: a database of transfer RNA genes detected in genomic sequence. Nucleic Acids Res 37: D93–D97. 10.1093/nar/gkn787 18984615 PMC2686519

[RNA077701LAKC5] Chan PP, Lowe TM. 2019. tRNAscan-SE: searching for tRNA genes in genomic sequences. Methods Mol Biol 1962: 1–14. 10.1007/978-1-4939-9173-0_1 31020551 PMC6768409

[RNA077701LAKC6] Cheloufi S, Dos Santos CO, Chong MMW, Hannon GJ. 2010. A dicer-independent miRNA biogenesis pathway that requires Ago catalysis. Nature 465: 584–589. 10.1038/nature09092 20424607 PMC2995450

[RNA077701LAKC7] Chen C, Ridzon DA, Broomer AJ, Zhou Z, Lee DH, Nguyen JT, Barbisin M, Xu NL, Mahuvakar VR, Andersen MR, 2005. Real-time quantification of microRNAs by stem-loop RT-PCR. Nucleic Acids Res 33: e179. 10.1093/nar/gni178 16314309 PMC1292995

[RNA077701LAKC8] Chen Q, Yan M, Cao Z, Li X, Zhang YY, Shi J, Feng GHG, Peng H, Zhang X, Zhang YY, 2016. Sperm tsRNAs contribute to intergenerational inheritance of an acquired metabolic disorder. Science 351: 397–400. 10.1126/science.aad7977 26721680

[RNA077701LAKC9] Cole C, Sobala A, Lu C, Thatcher SR, Bowman A, Brown JWS, Green PJ, Barton GJ, Hutvagner G. 2009. Filtering of deep sequencing data reveals the existence of abundant dicer-dependent small RNAs derived from tRNAs. RNA 15: 2147–2160. 10.1261/rna.1738409 19850906 PMC2779667

[RNA077701LAKC10] Darty K, Denise A, Ponty Y. 2009. VARNA: interactive drawing and editing of the RNA secondary structure. Bioinformatics 25: 1974–1975. 10.1093/bioinformatics/btp250 19398448 PMC2712331

[RNA077701LAKC11] Diederichs S, Haber DA. 2007. Dual role for argonautes in microRNA processing and posttranscriptional regulation of microRNA expression. Cell 131: 1097–1108. 10.1016/j.cell.2007.10.032 18083100

[RNA077701LAKC12] Du MZ, Wei W, Qin L, Liu S, Zhang AY, Zhang Y, Zhou H, Guo FB. 2017. Co-adaption of tRNA gene copy number and amino acid usage influences translation rates in three life domains. DNA Res 24: 623–633. 10.1093/dnares/dsx030 28992099 PMC5726483

[RNA077701LAKC13] Duret L. 2000. tRNA gene number and codon usage in the *C. elegans* genome are co-adapted for optimal translation of highly expressed genes. Trends Genet 16: 287–289. 10.1016/S0168-9525(00)02041-2 10858656

[RNA077701LAKC14] Eggertsson G, Soll D. 1988. Transfer ribonucleic acid-mediated suppression of termination codons in *Escherichia coli*. Microbiol Rev 52: 354–374. 10.1128/MR.52.3.354-374.1988 3054467 PMC373150

[RNA077701LAKC15] Ghildiyal M, Xu J, Seitz H, Weng Z, Zamore PD. 2010. Sorting of *Drosophila* small silencing RNAs partitions microRNA* strands into the RNA interference pathway. RNA 16: 43–56. 10.1261/rna.1972910 19917635 PMC2802036

[RNA077701LAKC16] Ginestet C. 2011. ggplot2: elegant graphics for data analysis. J R Stat Soc Ser A 174: 245–246. 10.1111/j.1467-985X.2010.00676_9.x

[RNA077701LAKC17] Goodarzi H, Liu X, Nguyen HCB, Zhang S, Fish L, Tavazoie SF. 2015. Endogenous tRNA-derived fragments suppress breast cancer progression via YBX1 displacement. Cell 161: 790–802. 10.1016/j.cell.2015.02.053 25957686 PMC4457382

[RNA077701LAKC18] Griffiths-Jones S, Saini HK, van Dongen S, Enright AJ. 2008. miRBase: tools for microRNA genomics. Nucleic Acids Res 36: D154–D158. 10.1093/nar/gkm952 17991681 PMC2238936

[RNA077701LAKC19] Grohme MA, Schloissnig S, Rozanski A, Pippel M, Young GR, Winkler S, Brandl H, Henry I, Dahl A, Powell S, 2018. The genome of Schmidtea mediterranea and the evolution of core cellular mechanisms. Nature 554: 56–61. 10.1038/nature25473 29364871 PMC5797480

[RNA077701LAKC20] Guzzi N, Cieśla M, Ngoc PCT, Lang S, Arora S, Dimitriou M, Pimková K, Sommarin MNE, Munita R, Lubas M, 2018. Pseudouridylation of tRNA-derived fragments steers translational control in stem cells. Cell 173: 1204–1216.e26. 10.1016/j.cell.2018.03.008 29628141

[RNA077701LAKC21] Harigaya Y, Parker R. 2017. The link between adjacent codon pairs and mRNA stability. BMC Genomics 18: 364. 10.1186/s12864-017-3749-8 28486986 PMC5424319

[RNA077701LAKC22] Hatfield D, Lee BJ, Smith DWE, Oroszlan S. 1990. Role of nonsense, frameshift, and missense suppressor tRNAs in mammalian cells. In Progress in molecular and subcellular biology (eds. Jeanteur P, ), pp. 115–146. Springer, Berlin, Heidelberg.

[RNA077701LAKC23] Higgs PG, Ran W. 2008. Coevolution of codon usage and tRNA genes leads to alternative stable states of biased codon usage. Mol Biol Evol 25: 2279–2291. 10.1093/molbev/msn173 18687657

[RNA077701LAKC24] Hiraoka Y, Kawamata K, Haraguchi T, Chikashige Y. 2009. Codon usage bias is correlated with gene expression levels in the fission yeast *Schizosaccharomyces pombe*. Genes Cells 14: 499–509. 10.1111/j.1365-2443.2009.01284.x 19335619

[RNA077701LAKC25] Hoehener C, Hug I, Nowacki M. 2018. Dicer-like enzymes with sequence cleavage preferences. Cell 173: 234–247.e7. 10.1016/j.cell.2018.02.029 29551264 PMC5871716

[RNA077701LAKC26] Honda S, Loher P, Shigematsu M, Palazzo JP, Suzuki R, Imoto I, Rigoutsos I, Kirino Y. 2015. Sex hormone-dependent tRNA halves enhance cell proliferation in breast and prostate cancers. Proc Natl Acad Sci 112: E3816–E3825. 10.1073/pnas.1510077112 26124144 PMC4517238

[RNA077701LAKC27] Ivanov P, Emara MM, Villen J, Gygi SP, Anderson P. 2011. Angiogenin-induced tRNA fragments inhibit translation initiation. Mol Cell 43: 613–623. 10.1016/j.molcel.2011.06.022 21855800 PMC3160621

[RNA077701LAKC28] Jehn J, Treml J, Wulsch S, Ottum B, Erb V, Hewel C, Kooijmans RN, Wester L, Fast I, Rosenkranz D. 2020. 5′ tRNA halves are highly expressed in the primate hippocampus and might sequence-specifically regulate gene expression. RNA 26: 694–707. 10.1261/rna.073395.119 32144192 PMC7266158

[RNA077701LAKC29] Karaiskos S, Naqvi AS, Swanson KE, Grigoriev A. 2015. Age-driven modulation of tRNA-derived fragments in *Drosophila* and their potential targets. Biol Direct 10: 51. 10.1186/s13062-015-0081-6 26374501 PMC4572633

[RNA077701LAKC30] Keam S, Hutvagner G. 2015. tRNA-derived fragments (tRFs): emerging new roles for an ancient RNA in the regulation of gene expression. Life 5: 1638–1651. 10.3390/life5041638 26703738 PMC4695841

[RNA077701LAKC31] Keam SP, Young PE, McCorkindale AL, Dang THY, Clancy JL, Humphreys DT, Preiss T, Hutvagner G, Martin DIK, Cropley JE, 2014. The human Piwi protein Hiwi2 associates with tRNA-derived piRNAs in somatic cells. Nucleic Acids Res 42: 8984–8995. 10.1093/nar/gku620 25038252 PMC4132735

[RNA077701LAKC32] Kim HK, Fuchs G, Wang S, Wei W, Zhang Y, Park H, Roy-Chaudhuri B, Li P, Xu J, Chu K, 2017. A transfer-RNA-derived small RNA regulates ribosome biogenesis. Nature 552: 57–62. 10.1038/nature25005 29186115 PMC6066594

[RNA077701LAKC33] Kim IV, Duncan EM, Ross EJ, Gorbovytska V, Nowotarski SH, Elliott SA, Sánchez Alvarado A, Kuhn CD. 2019. Planarians recruit piRNAs for mRNA turnover in adult stem cells. Genes Dev 33: 1575–1590. 10.1101/gad.322776.118 31537626 PMC6824462

[RNA077701LAKC34] Kretov DA, Walawalkar IA, Mora-Martin A, Shafik AM, Moxon S, Cifuentes D. 2020. Ago2-dependent processing allows miR-451 to evade the global microRNA turnover elicited during erythropoiesis. Mol Cell 78: 317–328.e6. 10.1016/j.molcel.2020.02.020 32191872 PMC7201373

[RNA077701LAKC35] Krishna S, Nair A, Cheedipudi S, Poduval D, Dhawan J, Palakodeti D, Ghanekar Y. 2013. Deep sequencing reveals unique small RNA repertoire that is regulated during head regeneration in Hydra magnipapillata. Nucleic Acids Res 41: 599–616. 10.1093/nar/gks1020 23166307 PMC3592418

[RNA077701LAKC36] Krishna S, Palakodeti D, Solana J. 2019a. Post-transcriptional regulation in planarian stem cells. Semin Cell Dev Biol 87: 69–78. 10.1016/j.semcdb.2018.05.013 29870807

[RNA077701LAKC37] Krishna S, Yim DG, Lakshmanan V, Tirumalai V, Koh JL, Park JE, Cheong JK, Low JL, Lim MJ, Sze SK, 2019b. Dynamic expression of tRNA-derived small RNAs define cellular states. EMBO Rep 20: e47789. 10.15252/embr.201947789 31267708 PMC6607006

[RNA077701LAKC38] Kumar L, Futschik ME. 2007. Mfuzz: a software package for soft clustering of microarray data. Bioinformation 2: 5–7. 10.6026/97320630002005 18084642 PMC2139991

[RNA077701LAKC39] Kumar P, Anaya J, Mudunuri SB, Dutta A. 2014. Meta-analysis of tRNA derived RNA fragments reveals that they are evolutionarily conserved and associate with AGO proteins to recognize specific RNA targets. BMC Biol 12: 78. 10.1186/s12915-014-0078-0 25270025 PMC4203973

[RNA077701LAKC40] Kuscu C, Kumar P, Kiran M, Su Z, Malik A, Dutta A. 2018. tRNA fragments (tRFs) guide Ago to regulate gene expression post-transcriptionally in a Dicer-independent manner. RNA 24: 1093–1105. 10.1261/rna.066126.118 29844106 PMC6049499

[RNA077701LAKC41] Lakshmanan V, Bansai D, Kulkarni J, Poduval D, Krishna S, Sasidharan V, Anand P, Seshasayee A, Palakodeti D. 2016. Genome-wide analysis of polyadenylation events in schmidtea mediterranea. G3 (Bethesda) 6: 3035–3048. 10.1534/g3.116.031120 27489207 PMC5068929

[RNA077701LAKC42] Langmead B, Trapnell C, Pop M, Salzberg S. 2009. Ultrafast and memory-efficient alignment of short DNA sequences to the human genome. Genome Biol 10: R25. 10.1186/gb-2009-10-3-r25 19261174 PMC2690996

[RNA077701LAKC43] Laslett D, Canback B. 2004. ARAGORN, a program to detect tRNA genes and tmRNA genes in nucleotide sequences. Nucleic Acids Res 32: 11–16. 10.1093/nar/gkh152 14704338 PMC373265

[RNA077701LAKC44] Lee SR, Collins K. 2005. Starvation-induced cleavage of the tRNA anticodon loop in *Tetrahymena thermophila*. J Biol Chem 280: 42744–42749. 10.1074/jbc.M510356200 16272149

[RNA077701LAKC45] Li W, Godzik A. 2006a. Cd-hit: a fast program for clustering and comparing large sets of protein or nucleotide sequences. Bioinformatics 22: 1658–1659. 10.1093/bioinformatics/btl158 16731699

[RNA077701LAKC46] Li W, Godzik A. 2006b. Cd-hit: a fast program for clustering and comparing large sets of protein or nucleotide sequences. Bioinformatics 22: 1658–1659. 10.1093/bioinformatics/btl158 16731699

[RNA077701LAKC47] Li Y-Q, Zeng A, Han X-S, Wang C, Li G, Zhang Z-C, Wang J-Y, Qin Y-W, Jing Q. 2011. Argonaute-2 regulates the proliferation of adult stem cells in planarian. Cell Res 21: 1750–1754. 10.1038/cr.2011.151 21894189 PMC3357986

[RNA077701LAKC48] Lowe TM, Eddy SR. 1997. tRNAscan-SE: a program for improved detection of transfer RNA genes in genomic sequence. Nucleic Acids Res 25: 955–964. 10.1093/nar/25.5.955 9023104 PMC146525

[RNA077701LAKC49] Luo S, He F, Luo J, Dou S, Wang Y, Guo A, Lu J. 2018. Drosophila tsRNAs preferentially suppress general translation machinery via antisense pairing and participate in cellular starvation response. Nucleic Acids Res 46: 5250–5268. 10.1093/nar/gky189 29548011 PMC6007262

[RNA077701LAKC50] Lyons SM, Achorn C, Kedersha NL, Anderson PJ, Ivanov P. 2016. YB-1 regulates tiRNA-induced Stress Granule formation but not translational repression. Nucleic Acids Res 44: 6949–6960. 10.1093/nar/gkw418 27174937 PMC5001593

[RNA077701LAKC51] Lyons SM, Gudanis D, Coyne SM, Gdaniec Z, Ivanov P. 2017. Identification of functional tetramolecular RNA G-quadruplexes derived from transfer RNAs. Nat Commun 8: 1127. 10.1038/s41467-017-01278-w 29066746 PMC5655342

[RNA077701LAKC52] Martin M. 2011. Cutadapt removes adapter sequences from high-throughput sequencing reads. EMBnet.journal 17: 10. 10.14806/ej.17.1.200

[RNA077701LAKC53] Martinez G, Choudury SG, Slotkin RK. 2017. TRNA-derived small RNAs target transposable element transcripts. Nucleic Acids Res 45: 5142–5152. 10.1093/nar/gkx103 28335016 PMC5605234

[RNA077701LAKC54] Mi S, Cai T, Hu Y, Chen Y, Hodges E, Ni F, Wu L, Li S, Zhou H, Long C, 2008. Sorting of small RNAs into Arabidopsis argonaute complexes is directed by the 5′ terminal nucleotide. Cell 133: 116–127. 10.1016/j.cell.2008.02.034 18342361 PMC2981139

[RNA077701LAKC55] Palakodeti D, Smielewska M, Lu Y-C, Yeo GW, Graveley BR. 2008. The PIWI proteins SMEDWI-2 and SMEDWI-3 are required for stem cell function and piRNA expression in planarians. RNA 14: 1174–1186. 10.1261/rna.1085008 18456843 PMC2390803

[RNA077701LAKC56] Peng H, Shi J, Zhang Y, Zhang H, Liao S, Li W, Lei L, Han C, Ning L, Cao Y, 2012. A novel class of tRNA-derived small RNAs extremely enriched in mature mouse sperm. Cell Res 22: 1609–1612. 10.1038/cr.2012.141 23044802 PMC3494397

[RNA077701LAKC57] Percudani R, Pavesi A, Ottonello S. 1997. Transfer RNA gene redundancy and translational selection in *Saccharomyces cerevisiae*. J Mol Biol 268: 322–330. 10.1006/jmbi.1997.0942 9159473

[RNA077701LAKC58] R Development Core Team. 2016. R: a language and environment for statistical computing. R Foundation for Statistical Computing, Vienna.

[RNA077701LAKC59] Reddien PW. 2018. The cellular and molecular basis for planarian regeneration. Cell 175: 327–345. 10.1016/j.cell.2018.09.021 30290140 PMC7706840

[RNA077701LAKC60] Reddien PW, Oviedo NJ, Jennings JR, Jenkin JC, Sánchez Alvarado A. 2005. Developmental biology: SMEDWI-2 is a PIWI-like protein that regulates planarian stem cells. Science 310: 1327–1330. 10.1126/science.1116110 16311336

[RNA077701LAKC61] Rozanski A, Moon HK, Brandl H, Martín-Durán JM, Grohme MA, Hüttner K, Bartscherer K, Henry I, Rink JC. 2019. PlanMine 3.0: improvements to a mineable resource of flatworm biology and biodiversity. Nucleic Acids Res 47: D812–D820. 10.1093/nar/gky1070 30496475 PMC6324014

[RNA077701LAKC62] Sarker G, Sun W, Rosenkranz D, Pelczar P, Opitz L, Efthymiou V, Wolfrum C, Peleg-Raibstein D. 2019. Maternal overnutrition programs hedonic and metabolic phenotypes across generations through sperm tsRNAs. Proc Natl Acad Sci 116: 10547–10556. 10.1073/pnas.1820810116 31061112 PMC6534971

[RNA077701LAKC63] Sasidharan V, Lu Y-C, Bansal D, Dasari P, Poduval D, Seshasayee A, Resch AM, Graveley BR, Palakodeti D. 2013a. Identification of neoblast- and regeneration-specific miRNAs in the planarian Schmidtea mediterranea. RNA 19: 1394–1404. 10.1261/rna.038653.113 23974438 PMC3854530

[RNA077701LAKC64] Sasidharan V, Lu YC, Bansal D, Dasari P, Poduval D, Seshasayee A, Resch AM, Graveley BR, Palakodeti D. 2013b. Identification of neoblast- and regeneration-specific miRNAs in the planarian Schmidtea mediterranea. RNA 19: 1394–1404. 10.1261/rna.038653.113 23974438 PMC3854530

[RNA077701LAKC65] Schorn AJ, Gutbrod MJ, LeBlanc C, Martienssen R. 2017. LTR-retrotransposon control by tRNA-derived small RNAs. Cell 170: 61–71.e11. 10.1016/j.cell.2017.06.013 28666125 PMC5551035

[RNA077701LAKC66] Sharma U, Conine CC, Shea JM, Boskovic A, Derr AG, Bing XY, Belleannee C, Kucukural A, Serra RW, Sun F, 2016a. Biogenesis and function of tRNA fragments during sperm maturation and fertilization in mammals. Science 351: 391–396. 10.1126/science.aad6780 26721685 PMC4888079

[RNA077701LAKC67] Sharma U, Conine CC, Shea JM, Boskovic A, Derr AG, Bing XY, Belleannee C, Kucukural A, Serra RW, Sun F, 2016b. Biogenesis and function of tRNA fragments during sperm maturation and fertilization in mammals. Science 351: 391–396. 10.1126/science.aad6780 26721685 PMC4888079

[RNA077701LAKC68] Sharma U, Sun F, Conine CC, Reichholf B, Kukreja S, Herzog VA, Ameres SL, Rando OJ. 2018. Small RNAs are trafficked from the epididymis to developing mammalian sperm. Dev Cell 46: 481–494.e6. 10.1016/j.devcel.2018.06.023 30057273 PMC6103849

[RNA077701LAKC69] Shibata N, Kashima M, Ishiko T, Nishimura O, Rouhana L, Misaki K, Yonemura S, Saito K, Siomi H, Siomi MC, 2016. Inheritance of a nuclear PIWI from pluripotent stem cells by somatic descendants ensures differentiation by silencing transposons in planarian. Dev Cell 37: 226–237. 10.1016/j.devcel.2016.04.009 27165555

[RNA077701LAKC70] Shivaprasad PV, Dunn RM, Santos BACM, Bassett A, Baulcombe DC. 2012. Extraordinary transgressive phenotypes of hybrid tomato are influenced by epigenetics and small silencing RNAs. EMBO J 31: 257–266. 10.1038/emboj.2011.458 22179699 PMC3261569

[RNA077701LAKC71] Sobala A, Hutvagner G. 2013. Small RNAs derived from the 5′ end of tRNAs can inhibit protein translation in human cells. RNA Biol 10: 553–563. 10.4161/rna.24285 23563448 PMC3710361

[RNA077701LAKC72] Solana J, Kao D, Mihaylova Y, Jaber-Hijazi F, Malla S, Wilson R, Aboobaker A. 2012. Defining the molecular profile of planarian pluripotent stem cells using a combinatorial RNAseq, RNA interference and irradiation approach. Genome Biol 13: R19. 10.1186/gb-2012-13-3-r19 22439894 PMC3439970

[RNA077701LAKC73] Sørensen MA, Kurland CG, Pedersen S. 1989. Codon usage determines translation rate in *Escherichia coli*. J Mol Biol 207: 365–377. 10.1016/0022-2836(89)90260-X 2474074

[RNA077701LAKC74] Speer J, Gehrke CW, Kuo KC, Waalkes TP, Borek E. 1979. tRNA breakdown products as markers for cancer. Cancer 44: 2120–2123. 10.1002/1097-0142(197912)44:6<2120::AID-CNCR2820440623>3.0.CO;2-6 509391

[RNA077701LAKC75] Stückemann T, Cleland JP, Werner S, Thi-Kim Vu H, Bayersdorf R, Liu SY, Friedrich B, Jülicher F, Rink JC. 2017. Antagonistic self-organizing patterning systems control maintenance and regeneration of the anteroposterior axis in planarians. Dev Cell 40: 248–263.e4. 10.1016/j.devcel.2016.12.024 28171748

[RNA077701LAKC76] Su Z, Kuscu C, Malik A, Shibata E, Dutta A. 2019a. Angiogenin generates specific stress-induced tRNA halves and is not involved in tRF-3-mediated gene silencing. J Biol Chem 294: 16930–16941. 10.1074/jbc.RA119.009272 31582561 PMC6851321

[RNA077701LAKC77] Su Z, Kuscu C, Malik A, Shibata E, Dutta A. 2019b. Angiogenin generates specific stress-induced tRNA halves and is not involved in tRF-3-mediated gene silencing. J Biol Chem 294: 16930–16941. 10.1074/jbc.RA119.009272 31582561 PMC6851321

[RNA077701LAKC78] Tåquist H, Cui Y, Ardell DH. 2007. TFAM 1.0: an online tRNA function classifier. Nucleic Acids Res 35: W350–W353. 10.1093/nar/gkm393 17591612 PMC1933168

[RNA077701LAKC79] Thompson DM, Lu C, Green PJ, Parker R. 2008. tRNA cleavage is a conserved response to oxidative stress in eukaryotes. RNA 14: 2095–2103. 10.1261/rna.1232808 18719243 PMC2553748

[RNA077701LAKC80] Tirumalai V, Prasad M, Shivaprasad P V. 2020. RNA blot analysis for the detection and quantification of plant micrornas. J Vis Exp 10.3791/61394. 10.3791/61394 32716394

[RNA077701LAKC81] Torres AG, Reina O, Attolini CSO, De Pouplana LR. 2019. Differential expression of human tRNA genes drives the abundance of tRNA-derived fragments. Proc Natl Acad Sci 116: 8451–8456. 10.1073/pnas.1821120116 30962382 PMC6486751

[RNA077701LAKC82] Tuorto F, Liebers R, Musch T, Schaefer M, Hofmann S, Kellner S, Frye M, Helm M, Stoecklin G, Lyko F. 2012. RNA cytosine methylation by Dnmt2 and NSun2 promotes tRNA stability and protein synthesis. Nat Struct Mol Biol 19: 900–905. 10.1038/nsmb.2357 22885326

[RNA077701LAKC83] Varenne S, Buc J, Lloubes R, Lazdunski C. 1984. Translation is a non-uniform process. Effect of tRNA availability on the rate of elongation of nascent polypeptide chains. J Mol Biol 180: 549–576. 10.1016/0022-2836(84)90027-5 6084718

[RNA077701LAKC84] Vidyanand S, Marepally S, Elliott SA, Baid S, Lakshmanan V, Nayyar N, Bansal D, Sánchez Alvarado A, Vemula PK, Palakodeti D. 2017. The *miR-124* family of microRNAs is critical for regeneration of the brain and visual system in the planarian *Schmidtea mediterranea*. Development 144: 3211–3223. 10.1242/dev.144758. 28807895 PMC5612250

[RNA077701LAKC85] Wagner DE, Wang IE, Reddien PW. 2011. Clonogenic neoblasts are pluripotent adult stem cells that underlie planarian regeneration. Science 332: 811–816. 10.1126/science.1203983 21566185 PMC3338249

[RNA077701LAKC86] Wang B. 2013. Base composition characteristics of mammalian mirnas. J Nucleic Acids 2013: 951570. 10.1155/2013/951570 23710337 PMC3595719

[RNA077701LAKC87] Wenemoser D, Lapan SW, Wilkinson AW, Bell GW, Reddien PW. 2012. A molecular wound response program associated with regeneration initiation in planarians. Genes Dev 26: 988–1002. 10.1101/gad.187377.112 22549959 PMC3347795

[RNA077701LAKC88] Wu Q, Bazzini AA. 2018. Systems to study codon effect on post-transcriptional regulation of gene expression. Methods 137: 82–89. 10.1016/j.ymeth.2017.11.006 29174654

[RNA077701LAKC89] Wu Q, Medina SG, Kushawah G, Devore ML, Castellano LA, Hand JM, Wright M, Bazzini AA. 2019. Translation affects mRNA stability in a codon-dependent manner in human cells. Elife 8: e45396. 10.7554/eLife.45396 31012849 PMC6529216

[RNA077701LAKC90] Yamasaki S, Ivanov P, Hu GF, Anderson P. 2009. Angiogenin cleaves tRNA and promotes stress-induced translational repression. J Cell Biol 185: 35–42. 10.1083/jcb.200811106 19332886 PMC2700517

[RNA077701LAKC91] Yu CH, Dang Y, Zhou Z, Wu C, Zhao F, Sachs MS, Liu Y. 2015. Codon usage influences the local rate of translation elongation to regulate co-translational protein folding. Mol Cell 59: 744–754. 10.1016/j.molcel.2015.07.018 26321254 PMC4561030

