# Supplementary material for: Comprehensive annotation and characterization of planarian tRNA and tRNA-derived fragments (tRFs)
Source: RNA. 2021 Apr;27(4):477–95. doi: 10.1261/rna.077701.120 (PMC7962491; doi:10.1261/rna.077701.120)
Supplement: Supplemental Material [file supp_27_4_477__index.html]

Comprehensive annotation and characterization of planarian tRNA and tRNA-derived fragments (tRFs) — Supplemental Material 

# Comprehensive annotation and characterization of planarian tRNA and tRNA-derived fragments (tRFs)

## Supplemental Material

- Supplemental\_Figures\_S1-S8.pdf
- Supplemental\_Material\_extended.pdf
- Supplemental\_Tables\_S1-S12.zip
