# Supplementary material for: Comprehensive annotation and characterization of planarian tRNA and tRNA-derived fragments (tRFs)
Source: RNA. 2021 Apr;27(4):477–95. doi: 10.1261/rna.077701.120 (PMC7962491; doi:10.1261/rna.077701.120)
Supplement: Supplemental Material [file supp_077701.120_Supplemental_Figures_S1-S8.pdf]

Supplementary Figure S1

A

	tRNA genes	Genomic Loci
**20 Standard amino acids**	303	942
**UNDET tRNAs**	44	125
**Pseudo tRNAs**	110	305

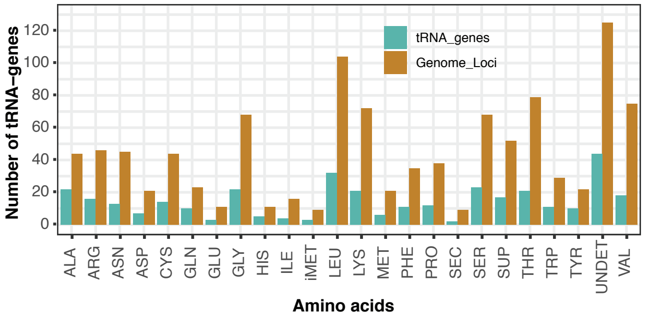

B

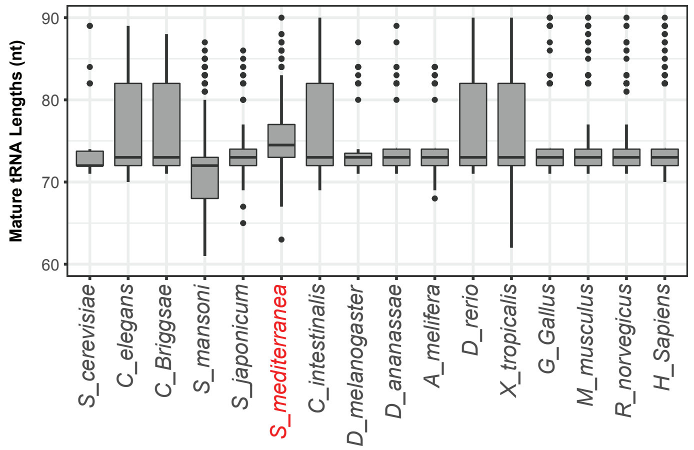

C

tRNA (anticodon) Number of Genes

Selenocysteine-tRNA Sec- TCA 2 **Undetermined-tRNA (Undet)** 44

First Base	Second Base: A				G				U				C				Third Base
A	AAA	6	10	(Phe)	AGA	7	2	(Ser)	AUA	2	1	(Tyr)	ACA	3	0	(Cys)	A
G	GAA	5	2		GGA	0	1		GUA	8	1		GCA	11	3		
U	UAA	7	4	(Leu)	UGA	7	2		UUA	11	11	Sup	UCA	3	0	Sup	
C	CAA	4	1		CGA	4	4		CUA	3	1		CCA	11	3	(Trp)	
A	AAG	4	2	(Leu)	AGG	3	2	(Pro)	AUG	0	0	(His)	ACG	2	1	(Arg)	G
G	GAG	0	0		GGG	1	0		GUG	5	2		GCG	1	0		
U	UAG	11	2		UGG	7	1		UUG	8	0	(Gln)	UCG	2	0		
C	CAG	6	1		CGG	1	1		CUG	2	1		CCG	1	1		
A	AAU	2	2	(Ile)	AGU	5	1	(Thr)	AUU	2	0	(Asn)	ACU	3	0	(Ser)	U
G	GAU	0	0		GGU	2	0		GUU	11	1		GCU	2	0		
U	UAU	2	0		UGU	10	2		UUU	15	9	(Lys)	UCU	6	0	(Arg)	
C	CAU	6<br>3	2<br>0	(Met)<br>(iMet)	CGT	4	0		CUU	6	2		CCU	4	1		
A	AAC	6	0	(Val)	AGC	6	0	(Ala)	AUC	0	0	(Asp)	ACC	0	0	(Gly)	C
G	GAC	0	0		GGC	4	3		GUC	7	3		GCC	10	4		
U	UAC	4	1		UGC	7	2		UUC	1	1	(Glu)	UCC	6	0		
C	CAC	8	0		CGC	5	0		CUC	2	1		CCC	6	0		

Figure S1: A) Table showing total number of tRNA genes predicted in planaria and the number of genomic loci for these tRNAs. **B)** Mature tRNA lengths across different species shows a median length of 72 nucleotides with planarian tRNAs showing a median of 75 nts. **C)** tRNA anticodon table decpicting all the anticodons and the number of tRNA genes identified for each anticodon (in green) and pseudogene (in grey).

Supplementary Figure S2

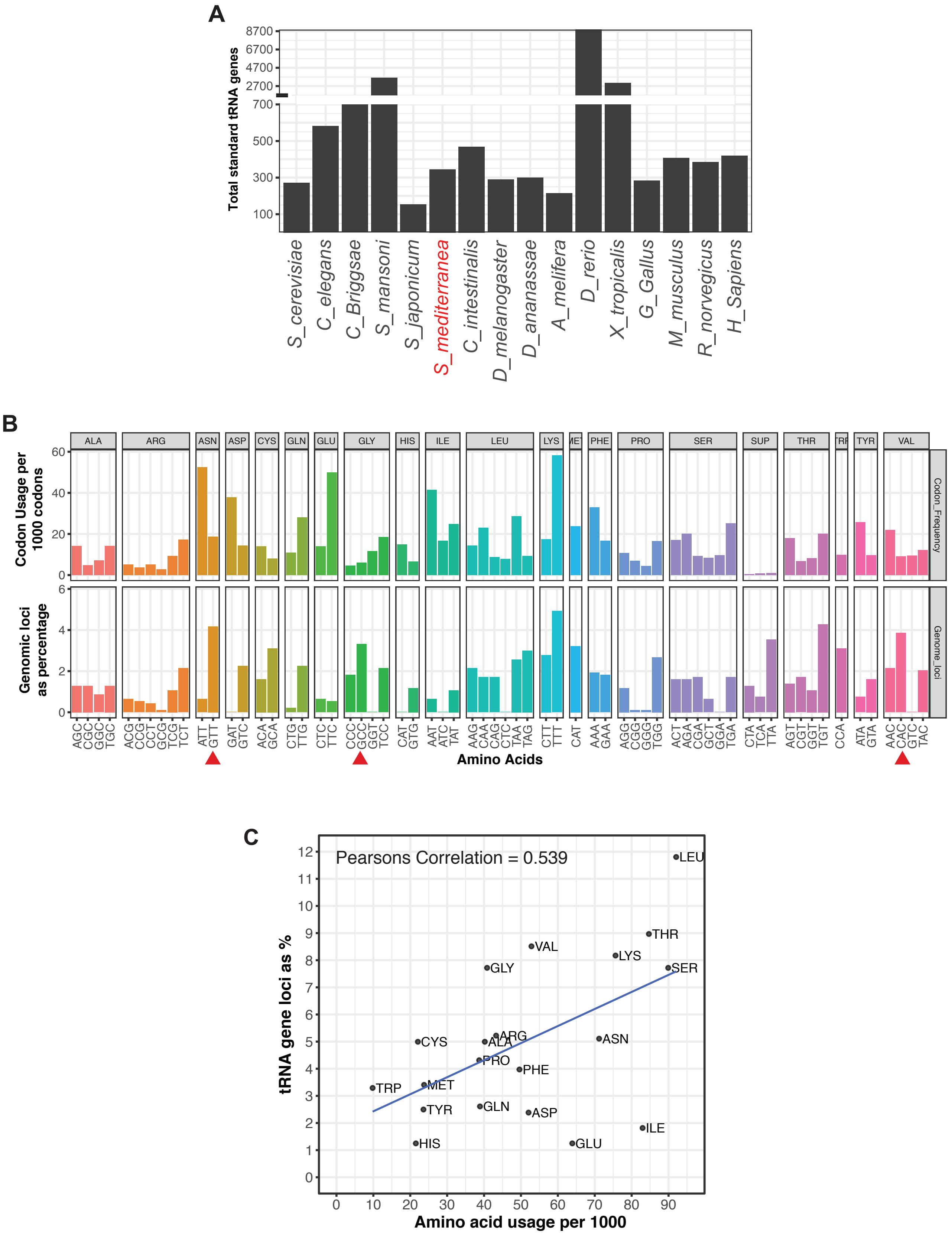

Figure S2: A) Number of tRNA genese for the standard 20 amino acids across the animal kingdom. Predicted planarian tRNA gene numbers are comparable across the species. **B)** Codon frequency across 1000 codons in the transcriptome and the abundace of its cognate anticodon tRNA in S.mediterranea genome. The red arrows examples of codon:anticon pairs that show opposite correlation. **C)** Correlation between tRNA gene copy number and amino acid usage in planarians.

Supplementary Figure S3

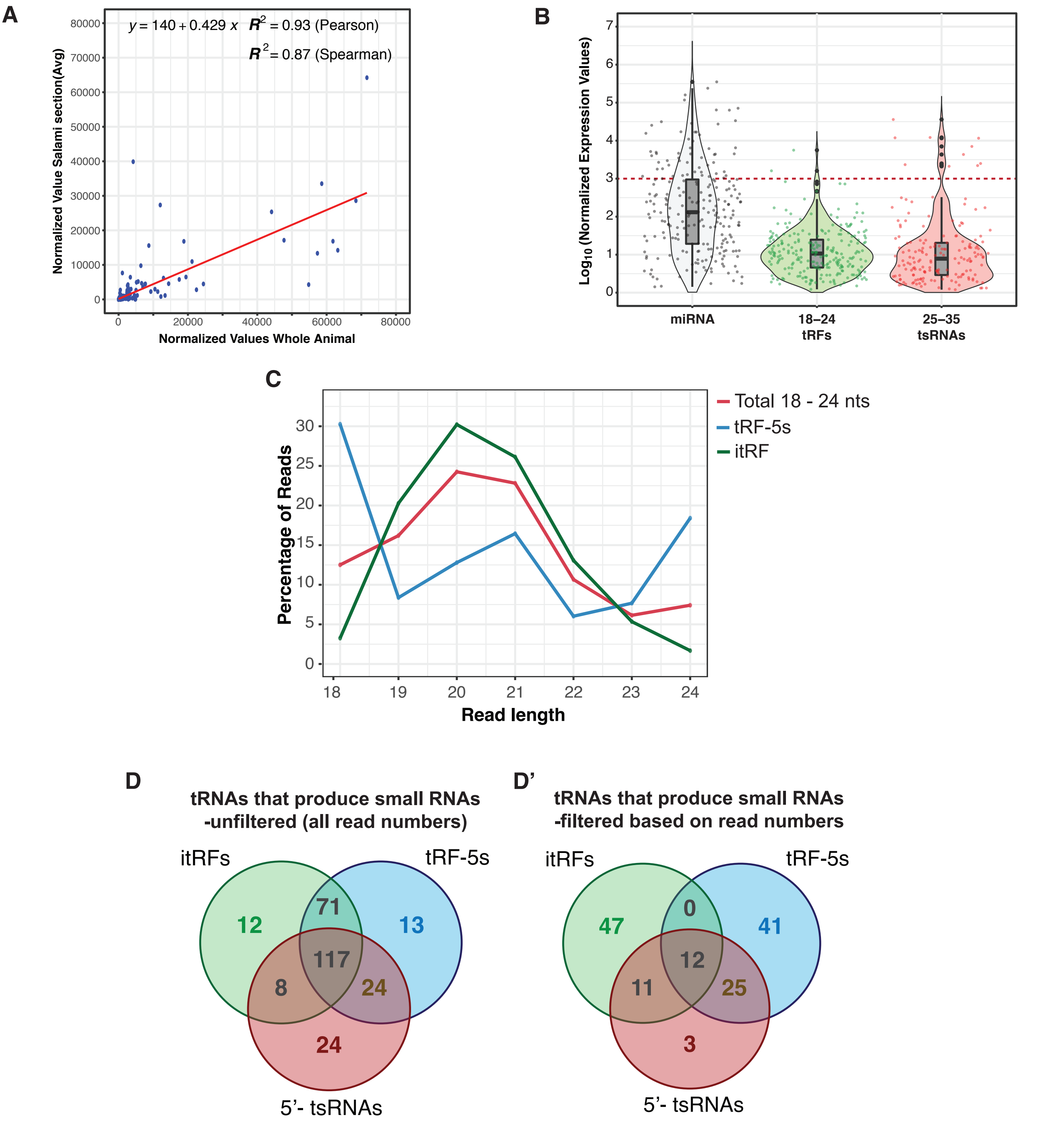

Figure S3: A) Correlation between small RNA reads mapping to tRNA in 'Uncut' and the cumulative of small RNA reads mapping to tRNA from Salami Sections. **B)** Normalised expression of all miRNAs compared to the reads mapping to tRNAs. The red dotted like indicates that the top 20 25-35 nt tsRNA species are expressed at higher levels than most miRNAs. **C)** Size distribution of itRFs and tRF-5s and the over all 18-24 reads mapping to tRNA. **D)** Venn diagram of tRNA that prodice itRFs, tRF-5s and 5'-tsRNAs. **D')** Venn diagram of tRNA that prodice itRFs, tRF-5s and 5'-tsRNAs using stringent criteria.

Supplementary Figure S4

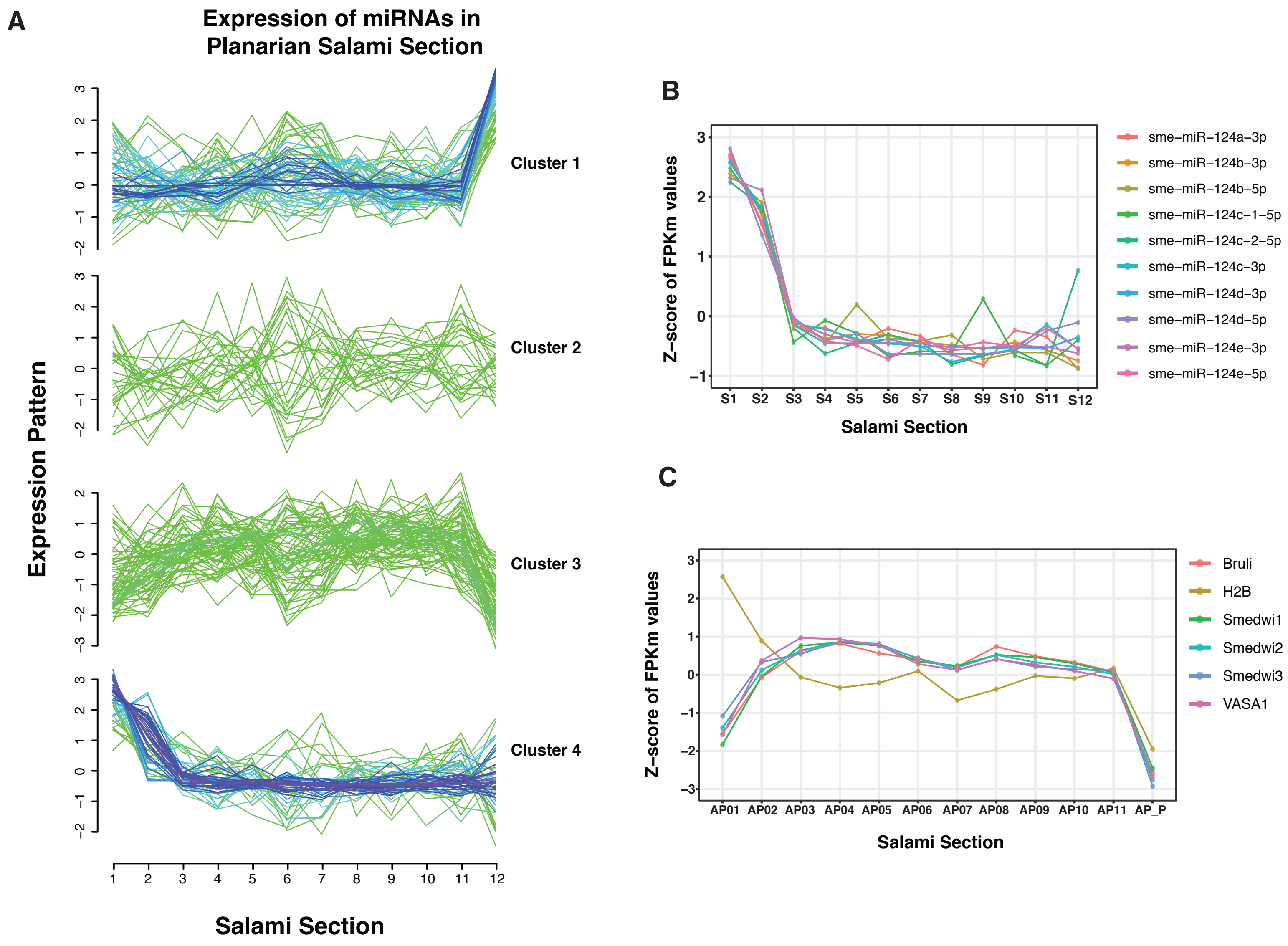

Figure S4: A) Expression clusters of miRNAs across planarian body axis identifies 4 clusters of expression. **B)** Expression of miR-124 family of miRNAs that are expressed in the brain shoes enrichments in the anterior sections of planaria. **C**) Expression of transcripts expressed in neoblasts across the different salami sections.

Supplementary Figure S5

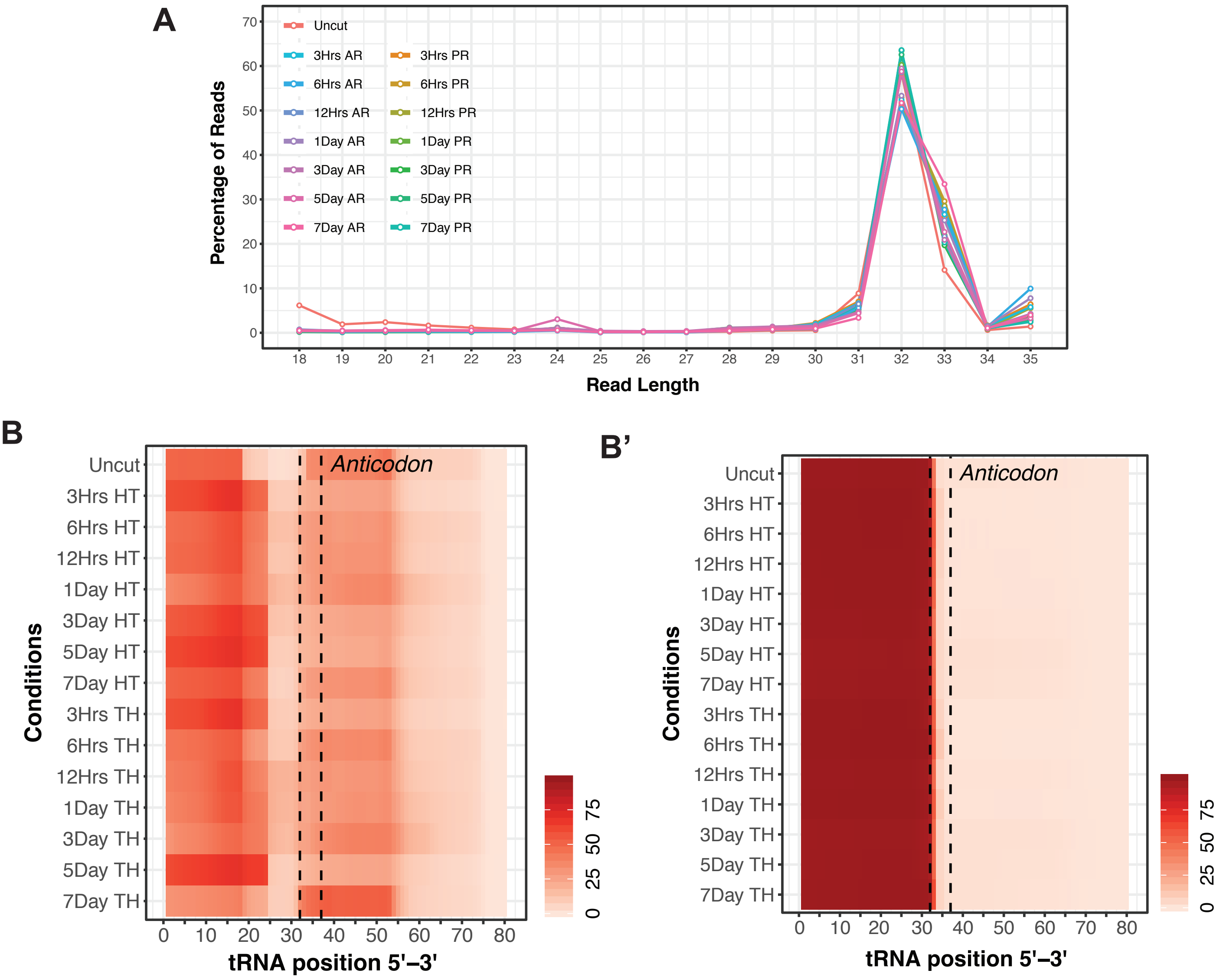

Figure S5: A) Size distribution of 18-35 nt small RNA reads that map to planarian tRNAs across regeneration timepoints. **B)** Perbase coverage of 18-24 nts of small RNA reads mapped to the tRNA length identifies two species of tRNA fragments - itRFs and tRF-5s. **B')** Perbase coverage of 25-35 nts of small RNA reads mapped to the tRNA length shows reads arise only from the 5' arm of tRNA.

Supplementary Figure S6

A

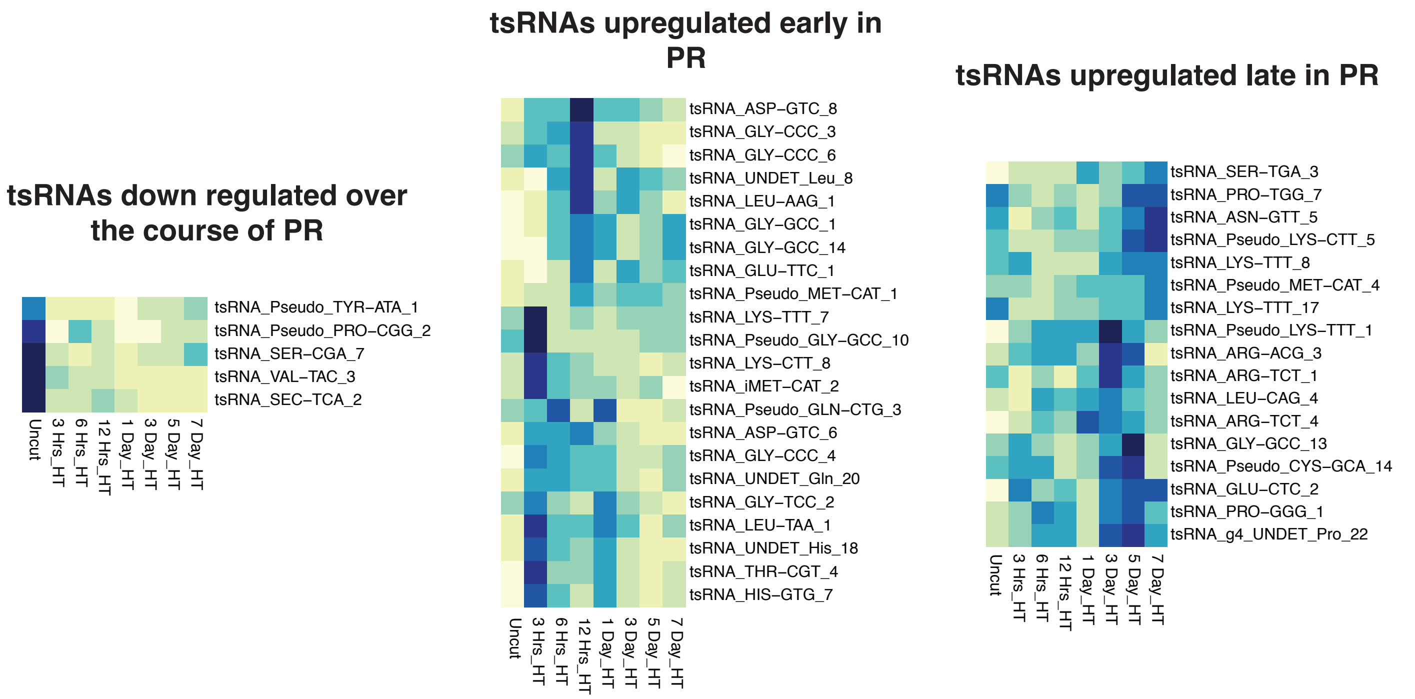

A’

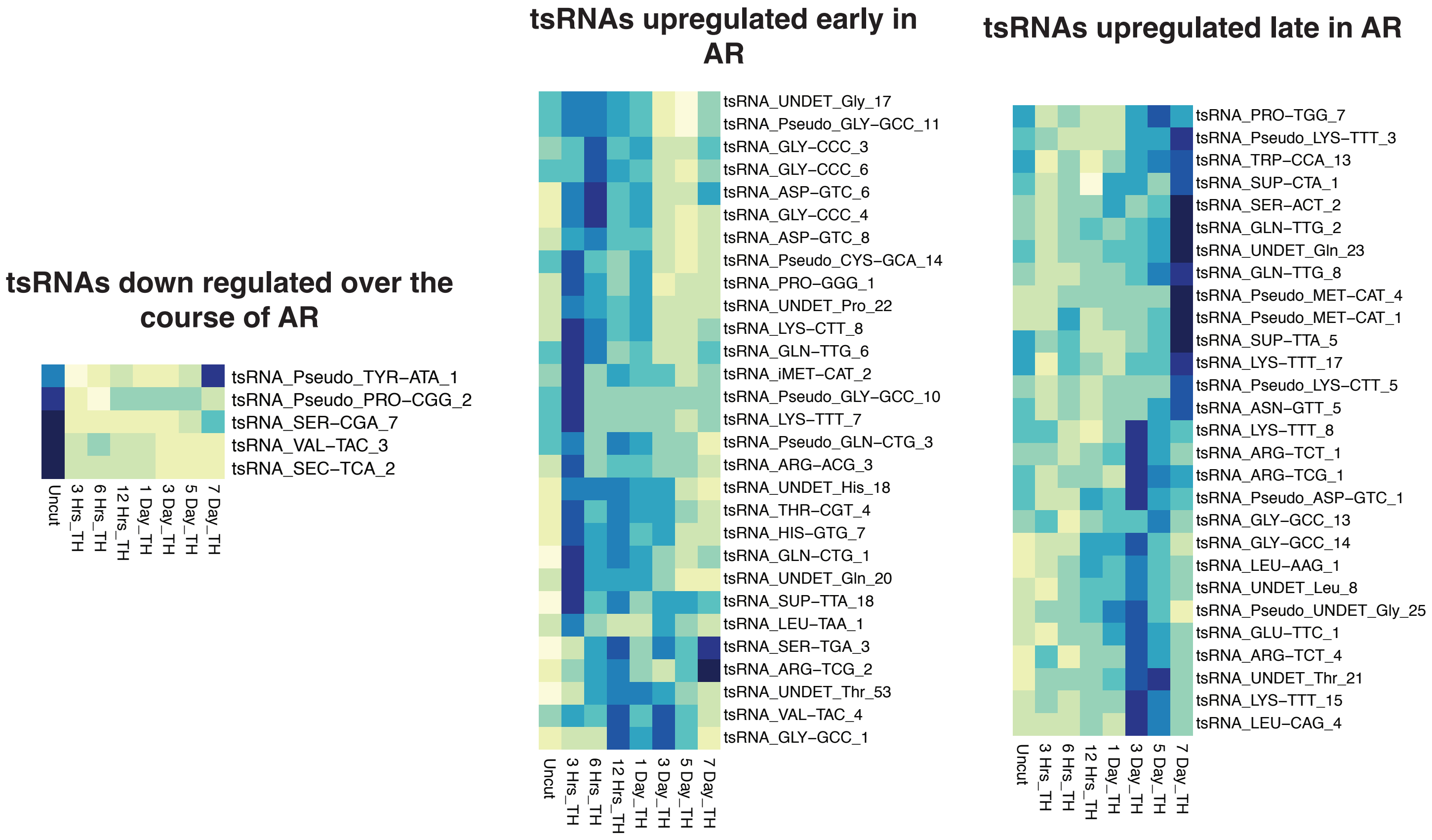

B

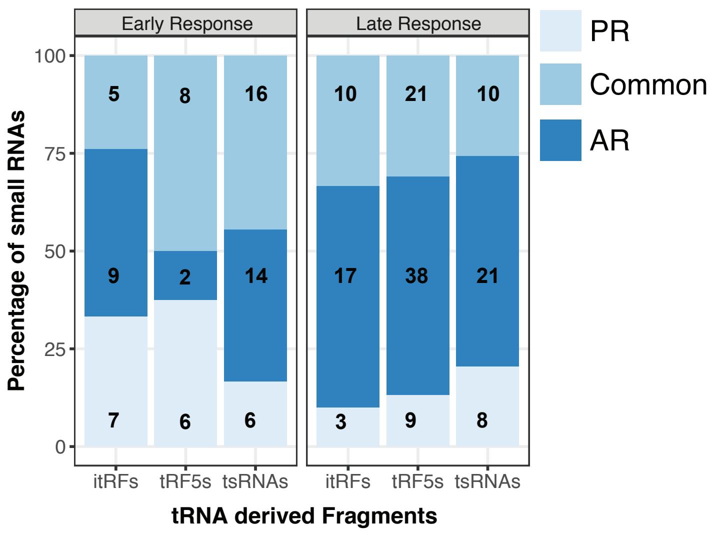

Figure S6: A) Heatmaps of 5’ tsRNAs during posterior regenerating (PR). **A’)** Heatmaps of 5’ tsRNAs in anterior regenerating worms (AR). **B)** Percentage of tRNA-derived fragments that are common and unique in early (3hrs -1day) and late (3days - 7days) regeneration of head (HT)and tail fragments (TH).

Supplementary Figure S7

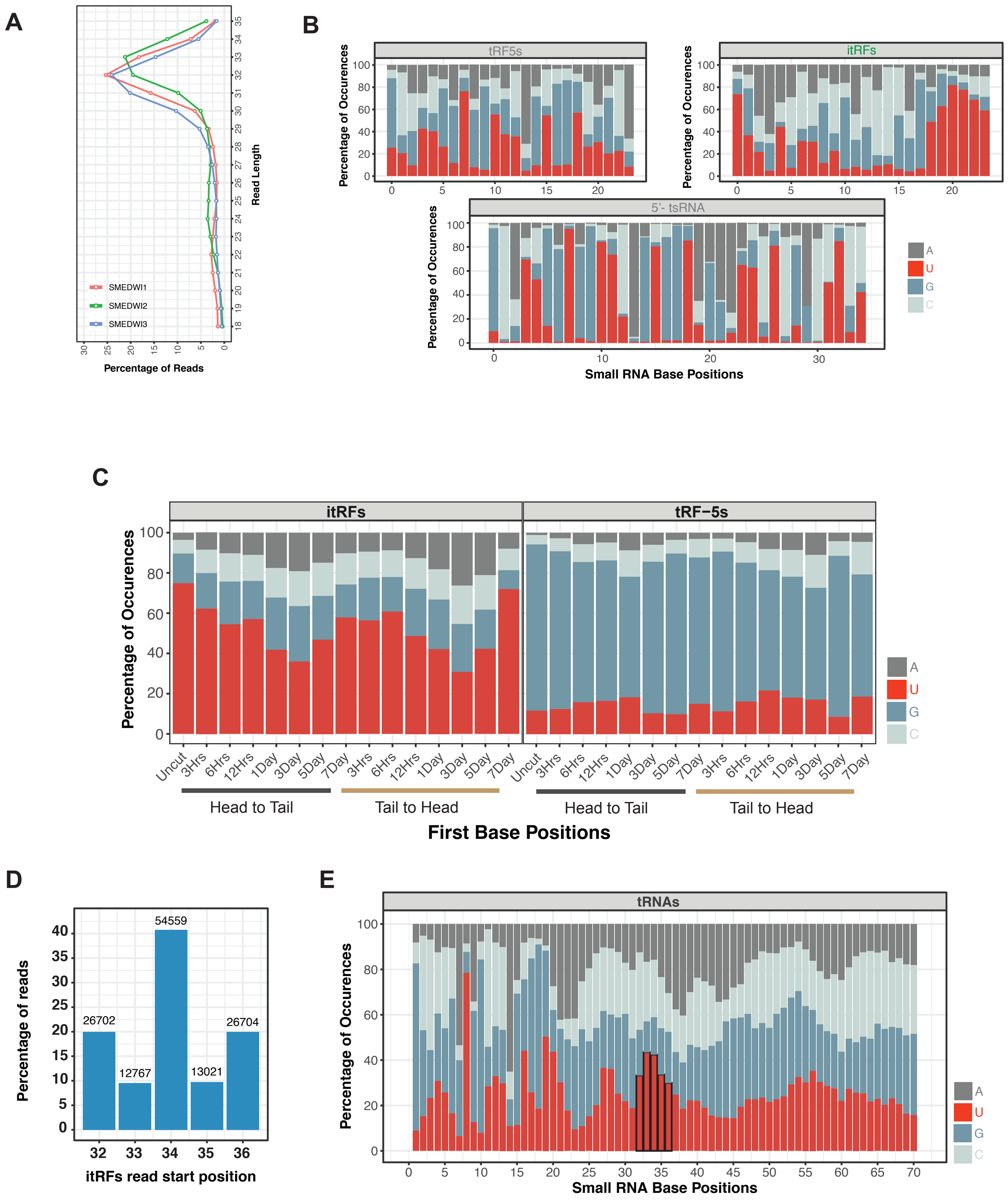

Figure S7: A) Size distribution of reads associating with SMEDWI-1, -2 and -3 that map to tRNAs. **B)** Base preference for the first base of all itRfs and tRF-5s during regeneration timepoints. **C)** Base preference across the length of tRNA-derived fragments identified in the study. **D)** Start positions of itRF fragments on the respective tRNAs from which they are processed. **E)** Base preference for the positions (from which itRFs are processed) across all tRNAs.

Supplementary Figure S8

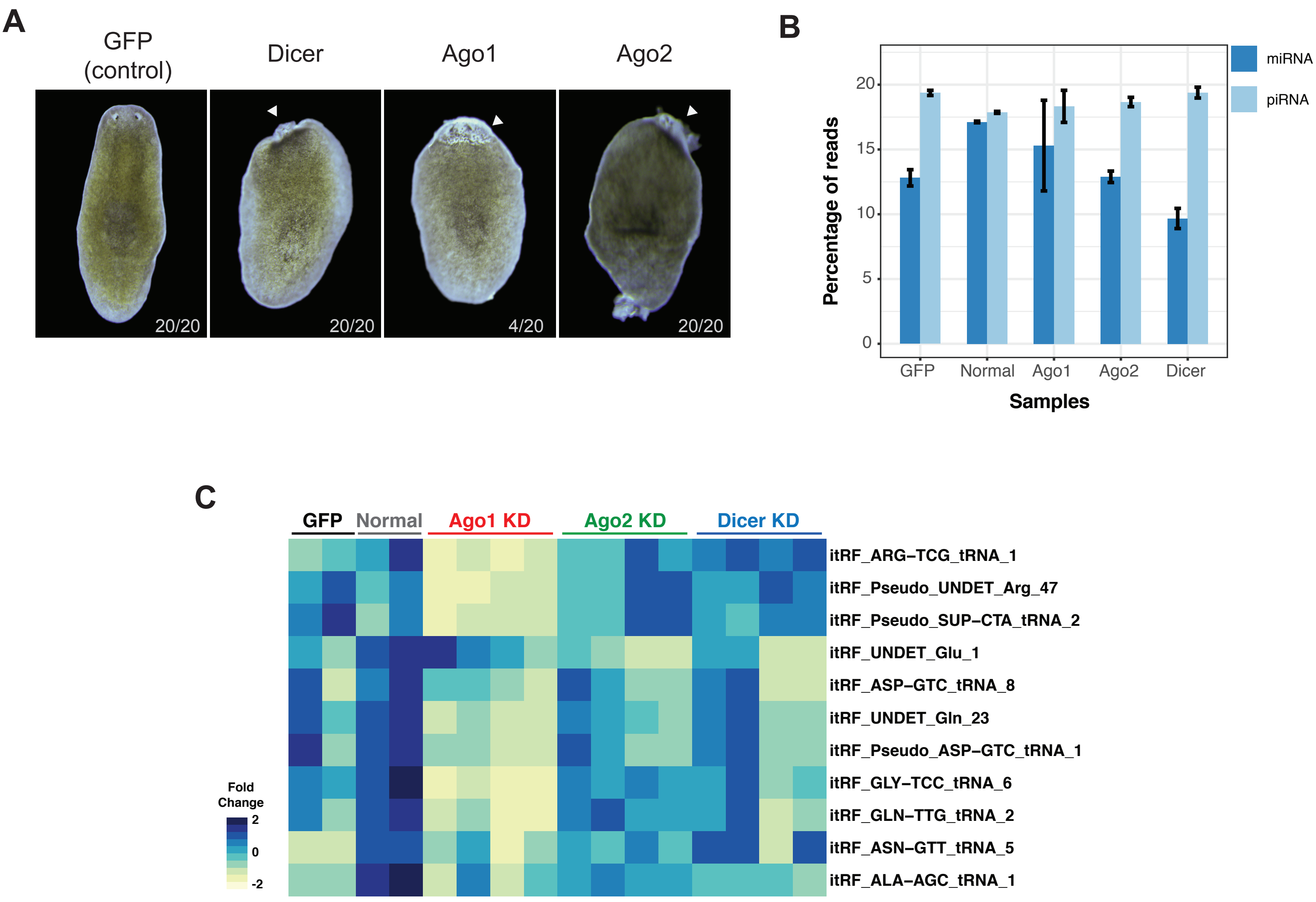

Figure S8: A) Phenotypes observed in planarians upon Ago1, Ago2 and Dicer Knockdown experiments. Inset shows then number of animals that showed phenotype out of the total animals tested (20). The arrow-heads depict head regression, lesions or lysis. **B)** miRNA and piRNA levels observed in different knockdown conditions. **C)** Expression of top 10 expressed itRFs in various knockdown conditions tested. Ago1 KD showed a decrease in the itRFs.
